# Uncovering injury-specific proteomic signatures and neurodegenerative risks in single and repetitive traumatic brain injury

**DOI:** 10.1038/s41392-025-02286-9

**Published:** 2025-06-23

**Authors:** Sarah Mantash, Soulaimane Aboulouard, Hassan Dakik, Yanis Zirem, Lydia Ziane-Chaouche, Ali Nehme, Khalil Mallah, Marya El-Kurdi, Naify Ramadan, Isabelle Fournier, Kazem Zibara, Firas Kobeissy, Michel Salzet

**Affiliations:** 1 Department of Experimental pathology, Immunology and Microbiology, Faculty of Medicine, American University of Beirut, Beirut, Lebanon; 2 Laboratoire Protéomique, Réponse Inflammatoire et Spectrométrie de Masse (PRISM), Univ. Lille, Inserm, CHU Lille, U1192, Lille, France; 3 Research Institute of the McGill University Health Centre, Montreal, QC Canada; 4 Department of Biochemistry and Molecular Genetics, Faculty of Medicine, American University of Beirut, Beirut, Lebanon; 5 Department of Pharmacology and Immunology, Medical University of South Carolina, Charleston, SC USA; 6 Center for Neurotrauma, Multiomics & Biomarkers (CNMB); Department of Neurobiology and Neuroscience Institute, Morehouse School of Medicine (MSM), Atlanta, GA USA

**Keywords:** Regeneration and repair in the nervous system, Predictive markers, Bioinformatics, Molecular engineering

## Abstract

Traumatic brain injury (TBI) is a major public health concern associated with an increased risk of neurodegenerative diseases including Alzheimer’s disease (AD), Parkinson’s disease (PD), and chronic traumatic encephalopathy, yet the underlying molecular mechanisms in repetitive TBI remain poorly defined. This study investigates proteomic and behavioral changes following single and repetitive mild TBI in a mouse model, focusing on molecular alterations in the cortex and hippocampus across acute (48 h) and subacute (1 week) stages. Using shotgun proteomics and bioinformatics approaches, including weighted gene co-expression network analysis (WGCNA) and machine learning, we analyzed the proteomic landscapes of TBI-affected brain regions including the hippocampus and the cortex. We assessed motor and cognitive outcomes at 2-, 7-, and 30-days post-injury to explore functional impairments associated with observed molecular changes. Our findings reveal spatio-temporal injury- and time-specific proteomic changes, with a single TBI promoting neuroprotective and repair mechanisms, while repetitive TBI exacerbating neuronal damage and synaptic deficits in the hippocampus. Key deregulated proteins, including *Apoa1*, *ApoE*, *Cox6a1*, and *Snca*, were linked to neurodegenerative pathways, suggesting molecular connections between TBI and diseases like AD and PD. Behavioral assessments indicated that repetitive TBI significantly impaired motor and cognitive functions, with recovery in motor function by day 30, whereas cognitive deficits persisted. This study provides a detailed analysis of the proteomic and behavioral consequences of TBI, identifying molecular networks as potential biomarkers or therapeutic targets for mitigating long-term cognitive decline associated with repetitive head trauma. These findings underscore the importance of mitochondrial and synaptic integrity in TBI response and suggest that targeting these pathways could reduce neurodegenerative risk following repetitive TBI.

## Introduction

Traumatic brain injury (TBI) is a serious neurological disorder that presents a significant health burden worldwide, with no widely effective pharmacological treatment available. TBI is induced by mechanical impacts on the brain, resulting in both immediate (primary) and delayed (secondary) cascades of neuropathological damage that can propagate to other central nervous system regions.^1,2^ Key neuropathological features of TBI include oxidative stress, neuronal damage, neuroinflammation, and blood-brain barrier (BBB) disruption.^3^ These effects collectively contribute to the lasting cognitive, motor, and psychological impairments frequently observed in TBI patients, and they can increase susceptibility to neurodegenerative diseases, such as AD and PD.^4–6^

Oxidative stress is one of the primary drivers of neuronal dysfunction following TBI, as it disrupts mitochondrial function, promotes cerebral edema, and damages the BBB.^7–10^ Oxidative stress is further amplified by a progressive neuroinflammatory response, where free radicals and proinflammatory cytokines released by glial cells initiate a shift in microglial cells toward either a proinflammatory (M1) or anti-inflammatory (M2) phenotype.^11^ Metabolically activated microglia are associated with the formation of brain edema and exacerbate neuroinflammation, thereby increasing the risk of chronic neural damage.^12–14^ These processes are intensified in cases of repetitive TBI, which are linked to more severe outcomes, including increased risk of memory loss, cognitive impairments, and the onset of neurodegenerative diseases.^15–19^ Despite extensive research on the effects of a single TBI, the molecular mechanisms and cellular pathways triggered by repetitive brain injuries remain incompletely understood as it simulates more military scenarios and sports-related concussions Although clinical profiles of repetitive TBI are well-documented, a comprehensive understanding of the cellular responses, particularly at different stages of injury, is limited. Repetitive injuries may uniquely disrupt brain function, particularly in regions such as the hippocampus, which plays a central role in learning and memory.^20–22^ Previous research has identified multiple proteomic changes following single TBIs, but these studies often overlook the dynamic and cumulative molecular effects associated with repetitive injury.^14,23–26^

Previous studies by our group have mapped proteomic changes in TBI models to correlate injury-site dynamics with the underlying biological processes.^13,14,23,27^ Using an experimental rat model of mild/moderate closed head injury, we revealed spatial and temporal overexpression of key proteins involved in neurodegenerative processes, such as synaptotagmins and glutamate decarboxylases, in the substantia nigra ipsilateral to the injury which implicated PD development.^14,23^ Our in vitro work also demonstrated that TBI-induced inflammation is associated with cellular responses indicative of neurodegenerative pathways, linking TBI with potential downstream neurological diseases.^14,23^ However, further investigations into how repetitive injuries impact proteomic and behavioral outcomes across time and brain regions are still underinvestigated.^14,23^

In the present study, we investigate the impact of repetitive TBI at both the proteomic and behavioral levels, focusing on different time points post-injury to characterize the associated molecular mechanisms. Male C57BL/6 mice were subjected to either a single or repetitive (three-hit) mild TBI, with proteomic profiling conducted at acute and subacute levels (48 h and 1-week post-injury) in both the cortex and hippocampus. Liquid chromatography-tandem mass spectrometry (LC-MS/MS) was used to assess the proteomic profile of the brain, while analyses of the protein-protein interaction (PPI) and the weighted gene co-expression network (WGCNA) provided insights into protein networks following injury. Behavioral tests were performed at 2, 7, and 30 days to assess motor and cognitive functions, capturing early acute (2 days), subacute (7 days), and chronic (30 days) effects of TBI. This study aims to provide a detailed proteomic and behavioral characterization of single and repetitive TBI, highlighting the distinct molecular responses and potential neurodegenerative risk associated with repetitive head trauma. By elucidating injury-specific and time-specific proteomic signatures, using complex and comprehensive multi-omics bioinformatic approaches, this research offers a robust foundation for identifying biomarkers and therapeutic targets to mitigate the long-term impact of repetitive TBI.

## Results

### Differentially expressed proteins

**Fig. 1:**
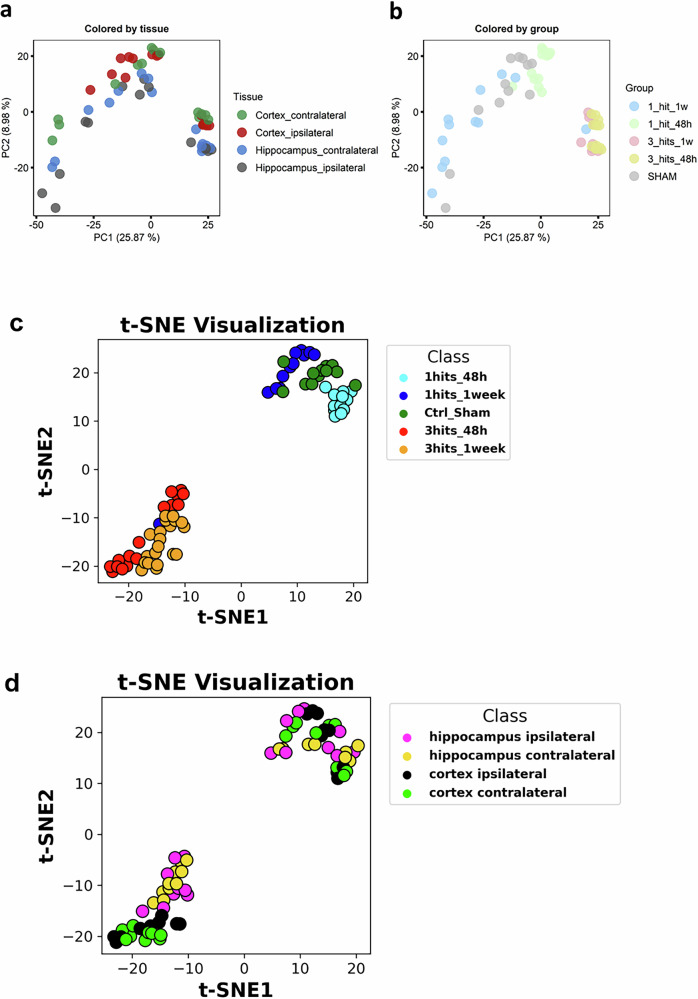
Unsupervised analysis of all proteins in the dataset. **a**, **b** Principal component analysis (PCA) shows the sample distribution along PC1 and PC2, which together account for ~35% of the total variance among samples. Left: Samples are colored by tissues. Right: Samples are colored by Hit-Time groups. **c**, **d** Unsupervised Machine learning using t-SNE algorithm

The proteomic approach of the TBI mouse model was performed with one (smTBI) or three hits (rmTBI) (Supplementary Fig. 1). Proteins were analyzed in the ipsilateral hemisphere (side of the injury) and contralateral hemisphere (opposite side of injury) parts of the hippocampus and the cortex after 48 h or 1-week post-injury in comparison to the sham group (Fig. 1).

Proteins extracted were processed using a “shotgun” analysis followed by label-free quantification (LFQ). Principal component analysis (PCA), an unsupervised linear model, was performed to describe the variance across all samples at different time points and injury conditions. The first principal component (PC1) explains 25.87% of the variance, while the second principal component (PC2) accounts for 8.98%, together explaining ~35% of the variability between samples (Fig. 1a, b). Samples categorized by tissue displayed overlapping clusters that were not distinctly separated (Fig. 1a). However, the number of hits served as a clear separator, with three-hit injuries (48 h and 1 week) clustering separately from other conditions and showing a positive correlation with PC1 (Fig. 1b).

**Fig. 2:**
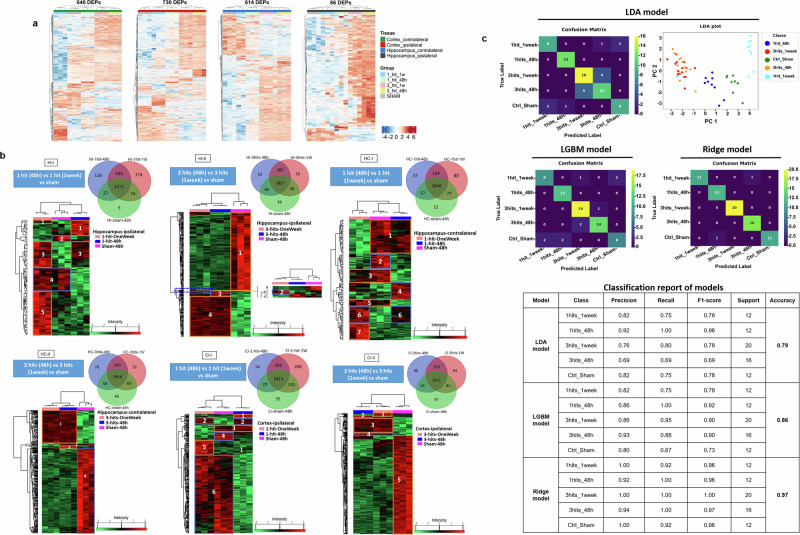
Heatmaps showing differentially-expressed proteins (DEPs) per tissue. DEPs were determined using a |log2(FC| > 1 and *q* < 0.05, comparing different Hit-Time groups to SHAM. In the heatmaps, red indicates high expression levels while blue represents low expression levels. Prior to clustering, the protein expression was centered and scaled. **a** Hierarchical clustering was conducted using Euclidean distance and average linkage to cluster the samples. Proteins were clustered using pearson correlation and average. More details about DEPs can be found in Supplementary Table 2. **b** Venn diagrams and heatmaps displaying the overlapped proteins that were deregulated in the hippocampus and cortex. Unique proteins in blue, red, or green, commonly altered proteins in the intersection of red, blue, and green. The Heat maps are based on the hierarchical clustering analysis corresponding to the detected proteins within the following conditions: sham, 1 hit, and 3 hits after 48 h or1 week post-injury taking the ipsilateral and contralateral hippocampus, and cortex regions (HI-I: Hippocampus 1 hit, HI-II: Hippocampus 3 hits, HC- I: Cortex 1 hit, HC-II: Hippocampus 3 hits). Distinct clusters are highlighted and assigned numbers from 1 to 7. Red corresponds to the upregulated proteins whereas green refers to the downregulated proteins. More details about the clusters can be found in Supplementary Table 3. **c** LDA plot and its classification report. Matrix confusion and classification report of LGBM model. Matrix confusion and classification report of the best and optimal model Ridge

Across all samples, 3,637 proteins were identified (Supplementary Table 1), with 2070 proteins significantly deregulated based on LFQ values. Heatmaps with hierarchical clustering revealed differentially expressed proteins (DEPs) among different hit-time points compared to the sham, with samples from similar hit conditions clustering together (Fig. 2). The number and identities of DEPs for each condition are provided in Supplementary Table 2. In most tissues, except for the contralateral hippocampus, protein expression profiles were similar between the 3-hit conditions at 48 h and 1 week. The largest number of differentially expressed proteins was observed in the ipsilateral cortex (730 DEPs), contralateral cortex (640 DEPs), and contralateral hippocampus (614 DEPs) compared to the sham, while the ipsilateral hippocampus exhibited fewer deregulated proteins (86 DEPs) (Fig. 2a). To gain additional insights into temporal patterns, we applied a clustering-based approach revealing distinct expression trajectories over time for each tissue (Fig. 2b).

To further explore sample distribution, we applied t-SNE, an unsupervised nonlinear model. Unlike PCA, which assumes linear relationships and fails to clearly separate tissue-specific clusters, t-SNE effectively captured complex, nonlinear patterns in the data. It provided a clearer separation between single mild TBI (SmTBI) and repeated mild TBI (RmTBI) based on the number of hits and time points (Fig. 1c). Additionally, when categorized by tissue, t-SNE outperformed PCA in distinguishing the hippocampus and cortex in the 3-hit condition (Fig. 1d). These differences likely reflect underlying biological variations between these regions, such as differential vulnerability to injury, distinct molecular responses, and region-specific proteomic changes.

### Proteomic analysis reveals hit and time-specific protein deregulation (or proteomic physiological stage)

Multiple comparisons along all experimental groups were conducted to determine the differentially expressed proteins in the hippocampus and the cortex after 1 hit or 3 hits, compared to sham at different time points (Fig. 2a and Supplementary Table 2).

Samples from the hippocampus ipsilateral region subjected to 1 hit displayed 3312 proteins out of which 89 were significantly deregulated and grouped into 5 clusters representing 5 Stages of deregulated proteins (Fig. 2b, HI-I). Stage 1 is represented by cluster 1 and characterized by proteins upregulated 48 h after injury, with expression partially decreasing after 1 week to similar conditions as the sham (Fig. 2b, HI-I). These proteins are involved in neurotransmission, vacuolar transport, mitotic cell cycle checkpoint, and stress-related response (Rims1, Chmp3, Klhl22, and HSPA1L). Stage 2 comprises cluster 2, proteins that are higher at baseline (sham) but fall after injury (48 h and 1-week post-injury). This cluster includes proteins implicated in cytoskeleton organization and mitochondrial NADH and ATP metabolic processes (Smarca2, Ndufa12, Atp6v1g1). Stage 3 is associated with proteins that are upregulated 48 h and 1 week postimpact and are involved in normal cell functions such as protein activation cascade, metabolic processes, mRNA stability (Arpp21, Dctn6, Rps15, Myef2), and inflammation (C1qa). Stage 4 may indicate partial restoration, with proteins returning toward their higher baseline (sham) abundance by 1-week post-injury. This Stage corresponds to cluster 4 which is involved mostly in axonal and cone growth, and regulation of apoptosis (Ptrh2, EPB41L3, Plxnb1). The last Stage 5 cluster 5 corresponds to proteins upregulated 1 week after injury. Proteins in this cluster are associated with neurotransmission, neurogenesis, and DNA repair in response to stress (Begain, Hprt1, Slc1a3, Tmem106b, Mcts1).

419 out of 3491 deregulated proteins were significantly identified from the hippocampus (Hp) ipsilateral tissues subjected to 3 hits. (Fig. 2b, HI-II). Hierarchal clustering performed on the 419 proteins showed 4 clusters corresponding to 4 Stages. Cluster 1 contains proteins that are highest at baseline (sham) and decline after injury (48 h and 1-week post-injury). This cluster is associated with the regulation of Leukocyte migration (as C1qbp, Rhoa), Ca2+ signaling and mitochondrial transport (Gja1, Mlc1), axonal transport, synaptic neurotransmission, and myelination (as Kif5c, snca, Nfasc), response to free radicles (sod1, Txnl1) and cellular respiration (Cox5a, Ndufb3). Stage 2 is characterized by proteomic pathways described in uninjured conditions and is represented by cluster 2 corresponding to a small group of proteins upregulated only at 1-week postimpact. These proteins are implicated in normal cellular functioning (Atl3, Cox7a2l). Stage 3 corresponds to proteins upregulated 48 h after injury, and then decreases after 1 week to similar conditions as the sham. These proteins associated with cluster 3 are involved in blood-associated processes such as platelet activation and aggregation, acute inflammatory response, and angiogenesis (Fga, Hp, Tinagl1). Cluster 4 which represents Stage 4 contains proteins that are upregulated at 48 h and 1-week post-injury. This cluster is associated with several ribosomal proteins (Rpl35, Rps15), cellular respiration (Cox6b1, Cox6a1), apoptosis (Aimp2, Mapk9), neurotransmission (Slc1a3, Syt12), neurogenesis (Myef2, Gpm6b), blood-related processes and inflammation (Mug1, Serpina3n, C1qa) and chromatin remodeling (Hdac11, Setd3).

The Hp contralateral samples showed 3265 proteins of which 122 were significantly altered upon 1 hit and grouped into 7 clusters (Fig. 2b, HC-I). Stage 1 is defined by proteins that are upregulated in the sham condition and remain elevated at 48 h following injury to later decrease after 1 week. This Stage is represented by cluster 1 which is linked to synaptic transmission and endocytosis (including Syt11, Nptxr), regulation of neurite morphogenesis (such as Kctd12, Dpysl3), and autophagy (Gnai3, Lamp2). Stage 2 is associated with cluster 2 cluster 2, proteins highest at baseline (sham) that drop after injury (48 h and 1 1-week post-injury). This Stage is correlated with the regulation of metabolic processes as cellular respiration and cell cycle/division (Slc7a11, Smc1a). Stage 3 represented by clusters 3 and 5 is associated with proteins upregulated at sham condition and 1-week post-injury referring to possible restoration of function. This Stage is involved mainly in the regulation of neuronal cell death and autophagy in response to injury, neurite development, and Ca2+ binding (Faim2, Arsb, S100a16, Cdkl5, Gap43). Stage 4 corresponding to cluster 4 is implicated in metabolic processes including cellular respiration (Atp6v1a, Nutf2) where proteins are upregulated 48 h after injury and partially downregulated after 1 week like the sham condition. Stage 5 is represented by cluster 6 which shows proteins upregulated at 48 h and 1-week post-injury. This cluster is correlated mainly with vesicular transport and morphogenesis (Tuba4a, Vamp4). Stage 6, represented by cluster7, corresponds to proteins that are exclusively upregulated 1-week post-injury and is associated with synaptic transmission and negative regulation of neuronal cell death (Serbp1, Gria2, Dpp8).

In the Hp contralateral region with 3 hits, 869 out of 3540 proteins were significant and grouped into 4 clusters representing 4 Stages (Fig. 2b, HC-II). Stage 1 features proteins already high at baseline (sham) that stay high at 48 h and decline by 1-week after injury. This Stage is associated with cellular apoptosis (Aimp2, Pea15, Mdh1), synaptic neurotransmission (Nptx1, Gria2), immune response (C1qa, Serpina1a, Gstp1), epigenetic regulation (Smyd5, Hdac11), and regulation of translation including ribosomal proteins such as: Rpl30, Rpl35. Stage 2 corresponds to a smaller group of proteins that are upregulated in sham condition and 48 h post-injury. This Stage is related to cluster 2 and is involved in response to oxidative stress and apoptosis (Hp, Cntfr, Pten). Stage 3 represents proteins that are upregulated at 48 h and then decrease 1-week post-injury similarly to the sham condition. This Stage includes proteins that are mainly associated with autophagy, platelet activation, and inflammation (Atg2b, Fgb, Serpina3k). The last Stage corresponds to proteins in cluster 4 that are upregulated in sham condition only and are associated with neurotransmission, apoptosis, regeneration, and protein transport (Homer1, Sod1, Rangap1, Ndufa7).

In the cortex, ipsilateral samples, 1 hit injury displayed 719 significant proteins out of 3524 with 6 clusters corresponding to 6 Stages (Fig. 2b, CI-I). Stage 1 represents proteins that are upregulated directly post-injury, i.e., 48 h, and subsequently restore their downregulated expression at 1 week. Cluster 1 is implicated mainly with cell cycle and DNA damage checkpoint (Klhl22, Fbxo6), regulation of translation, and synaptic neurotransmission (Adar, Homer3). Stage 2 corresponds to proteins upregulated 48 h and 1-week post-injury, represented by cluster 2. These proteins are involved in translation and protein homeostasis (Slc7a5, Rabgap1, Nop58). Stage 3 is represented are upregulated by cluster 3 where proteins are clustered at the sham condition and 48 h, then the expression is downregulated at 1-week post-injury. This Stage is associated with proteins associated with cellular respiration (Ndufaf2, Cox7a2l), synaptic neurotransmission (Actg1, Mapk10), and regulation of protein transport (Hspa8, Praf2, S100a13). Stage 4 corresponding to cluster 4 is associated with proteins that are upregulated in sham condition only and is involved in protein stability and binding (Ipo9, Pfdn2, Tom1), regulation of axonogenesis (Epha4, Cacna1a), cellular respiration (Ndufaf3, Ndufb9). Stage 5 is associated with cluster 5 and presents proteins that are upregulated only after 1 week of injury and implicated in synaptic translation and transmission (Epb41, Nrgn) as well as protein binding (Adam22, Slc22a23). Stage 6 is represented by cluster 6 where proteins whose abundance is high at baseline (sham), suppressed at 48 h, and re-elevated by 1 w, suggesting restoration. Cluster 6 contains a group of neuroprotective proteins such as heat shock-related proteins (Hspa2, Hspa9), FABP7, and Basp1 that are implicated in the initiation of axonal regeneration and remyelination. Other proteins were implicated in the regulation of neuronal apoptosis and cellular response to oxidative stress (Prdx1, Oxr1, Snca) as well as synaptic neurotransmission (Syngr3, Synj1, Nrxn1).

In the cortex ipsilateral samples with 3 hits, a larger number of 1310 out of 3561 proteins were significantly deregulated and were listed into 5 clusters representing 4 Stages (Fig. 2b, CI-II). Stage 1 is associated with cluster 1 and represents proteins that are upregulated only until 1 week post-injury. These proteins are involved in axonal growth and synaptic neurotransmission (Ncald, Ncam2, Stxbp1). Stage 2 corresponds to cluster 2, which consists of a small set of proteins high at baseline (sham) that remain elevated at 48 h and normalize by 1-week. Cluster 2 is correlated with neuronal proliferation and differentiation (S100b, Nhsl1), angiogenesis, and inflammation (Apoh, Ahsg). Stage 3 is represented by clusters 3 and 4 where proteins are upregulated at 48 h and 1 week after injury. Cluter3 and 4 are associated with cellular respiration (Ndufa11, Atp6v1a, Cox6a1), translation (Rps8, Mrps23, Eif2b5), lipid metabolism (Acsf3, Hacd3), apoptosis (Aimp2, Atg5, Aqp4), inflammation (Cd47, Serpina1a) and neurogenesis and synaptic transmission (Gpm6b, Nptx1). Stage 4, represented by cluster 5, is associated with a large group of proteins that are upregulated only in the sham condition and are downregulated 48 h and 1 week post-injury. Proteins downregulated in this cluster are mainly associated with axon guidance and neurogenesis (Nrn1, Nefm, Epha4, Sema4d), myelination and regeneration (Plp1, Mag), vesicle Trafficking and synaptic signaling (Syngap1, Syngr3, Snca). Other groups of proteins were deregulated in response to stress and are implicated in apoptosis (Api5, Aifm1), repair such as heat shock proteins (Hspa4, Hspa9), anti-oxidation (Nos1, Oxr1, Sod1), inflammation (Cd47, Serpinb1a), and cellular respiration (Cox5b, Ndufs6, Ndufa6). The number and identity of the deregulated proteins and clusters in each region are displayed in Supplementary Table 3.

Supervised learning analyses were conducted on the proteomic dataset. Out of the 24 models evaluated, the top three performers (Linear Discriminant Analysis (LDA), LightGBM (LGBM), and Ridge) were highlighted in Fig. 2c, with detailed classification reports and confusion matrices provided for each model. The 5-fold cross-validation scores and corresponding accuracies for LDA, LGBM, and Ridge were 0.7885 ± 0.151, 0.86 ± 0.0255, and 0.9714 ± 0.0349, respectively (Fig. 2c). Among these, Ridge was identified as the best-performing model, achieving an F1 score of up to 96% across all conditions, demonstrating an optimal balance between specificity and sensitivity. To gain insights into the predictions made by the Ridge classifier, the Local Interpretable Model-Agnostic Explanations (LIME) algorithm was employed. LIME identified specific proteins most discriminative for each condition, as detailed in Supplementary Fig. 2. The identified proteins listed in Supplementary Fig. 2 reveal potential associations with TBI and neurodegenerative diseases. Notably, Sirtuin-3 (sirt3), a mitochondrial enzyme, plays a key role in neuroprotection and mitigating neurodegenerative processes by regulating oxidative stress.^28^ The Potassium voltage-gated channel subfamily D member 3 (kcnd3) is linked to spinocerebellar ataxia, a motor coordination disorder, through mutations impacting neuronal excitability.^29^ Coq3, involved in coenzyme Q10 biosynthesis, highlights the importance of mitochondrial function, as deficiencies can lead to mitochondrial dysfunction, a hallmark in various neurodegenerative diseases.^30^ Other proteins, such as chmp7 and epha6, are involved in cellular pathways linked to membrane remodeling and neuronal development, respectively, and show associations with neurodegeneration when these pathways are disrupted.^31,32^ Additionally, Adar enzymes influence gene expression through RNA editing, and dysregulation has been observed in neurodegenerative conditions.^33^ Hspa1l, a heat shock protein, contributes to cellular stress responses and has been studied in the context of neurodegeneration.^34^ Lastly, Snap-25 is crucial for synaptic vesicle fusion and neurotransmitter release, with alterations linked to synaptic dysfunction in both TBI and neurodegenerative diseases.^35^ The comparison of LIME-identified proteins (Supplementary Fig. 2 and Supplementary Fig. 3) from proteomic data based on Data Dependent analysis (DDA) cross validated by Data independent analyses (DIA-NN) (Supplementary Fig. 4) from ND-related proteins (Table 1) reveals significant overlaps that underscore critical pathways impacted by TBI and linked to neurodegenerative disease risk. Key proteins such as *Cox6a1*, *Rpl35*, *Apoa1*, *Snca*, and *Tfam* are present in both datasets, highlighting mitochondrial dysfunction, synaptic integrity, and lipid dysregulation as central mechanisms affected by TBI. *Cox6a1* and *Tfam*, associated with mitochondrial health, emphasize the vulnerability of energy production pathways to repetitive TBI, which can lead to long-term neurodegeneration. Similarly, *Snca* and *Rpl35* indicate disruptions in synaptic function and protein synthesis processes crucial for cognitive resilience and cellular health. *Apoa1* reflects alterations in lipid transport, which may exacerbate neuroinflammation, a hallmark in both TBI and neurodegenerative conditions. The consistent identification of these proteins across LIME and proteomic analyses suggests their potential as reliable biomarkers for assessing TBI severity and neurodegenerative risk, while also presenting viable therapeutic targets. By integrating machine learning with proteomic data, this study illuminates molecular pathways that could guide future diagnostic and treatment strategies for TBI-induced neurodegeneration.

**Table 1:** Overlapping proteins for neurodegenerative diseases (Top 20)

		Cortex				Hippocampus			
		Contralateral		Ipsilateral		Contralateral		Ipsilateral	
		1H	3H	1H	3H	1H	3H	1H	3H
**PD**	48 h	-	Cox6a1	Ache- Ibl1	Cox6a1	-	Cox6a1- Apoa1	-	Cox6a1- Apoa1- Snca
	1 week	Tfam- Aco2- Gc	Cox6a1 -Tfam- Gc	Tfam	Cox6a1- Psap	-	Cox6a1- Gc	-	Cox6a1- Snca
**AD**	48 h	Capn1	Rpl35	Aqp4- Rpl35- Ache	Cox6a1- Aqp4- Rpl35- Epha4	Adam10	Cox6a1- Rpl35- Apoa1	Hspa1l	Cox6a1- C3- Rpl35- Apoa1- Ndufb3- Snca
	1 week	Nrgn- Tfam- Gc- Sod2	Cox6a1- Rpl35- Ndufa3- Tfam- Gc	Aqp4- Tfam- Nrgn	Cox6a1- Aqp4- Rpl35	-	Cox6a1- Rpl35- Gc	-	Cox6a1- Rpl35- Snca
**HD**	48 h	-	Ppp1r1b	Ache	-	-	-	-	Ndufb3
	1 week	Tfam	Tfam	Tfam	-	-	-	-	-
**ALS**	48 h	Rpl35	Rpl35	Aqp4- Rpl35	Ap4- Rpl35	-	Rpl35	-	Rpl35
	1 week	Tfam- Sod2	Rpl35- Tfam	Aqp4- Tfam	Ap4- Rpl35	-	Rpl35	-	Rpl35
**MS**	48 h	-	-	Ache	-	-	Apoa1	-	Apoa1
	1 week	Gc	Gc	-	-	-	Gc	-	-
**Epilepsy**	48 h	C1qa	-	Aqp4- Ache	Aqp4	C1qa	-	-	-
	1 week	Nrgn	-	Aqp4	Aqp4	-	C1qa	-	C1qa

### Proteomics analysis performed on the cortex and hippocampus reveals deregulation in ND-related proteins

Pairwise analysis conducted on tissues at 1 and 3 hits conditions at different time points each compared to sham revealed several proteins that overlapped with neurodegenerative disorders (ND) (Top 20 overlapping proteins are illustrated in Table 1 and detailed in Supplementary Table 3). Among the deregulated proteins, cytochrome c oxidase subunit 6 (Cox6a1), and synuclein alpha (snca) were reported to be involved in PD and AD. Other overlapping proteins were deregulated in multiple sclerosis (MS), PD, and AD including apolipoprotein A1 (Apoa1). Interestingly, calpain 1 (Capn1), Neurogranin (Nrgn), and ribosomal protein L35 (Rpl35) were reported to be implicated in AD. Furthermore, the transcription factor A, several NADH-ubiquinone oxidoreductases (Ndufa3, Ndufb3) were involved in AD and Huntington’s disease (HD). Interestingly, among the upregulated proteins, glutamate decarboxylase (GAD1) and apolipoprotein (ApoE) have been previously reported to be involved in PD and AD respectively although it was not among the top 20 overlapping genes. Notably, several deregulated proteins in our study were further associated with dementia (Snca and Gria4).

### Biological processes enrichment analysis across all conditions

**Fig. 3:**
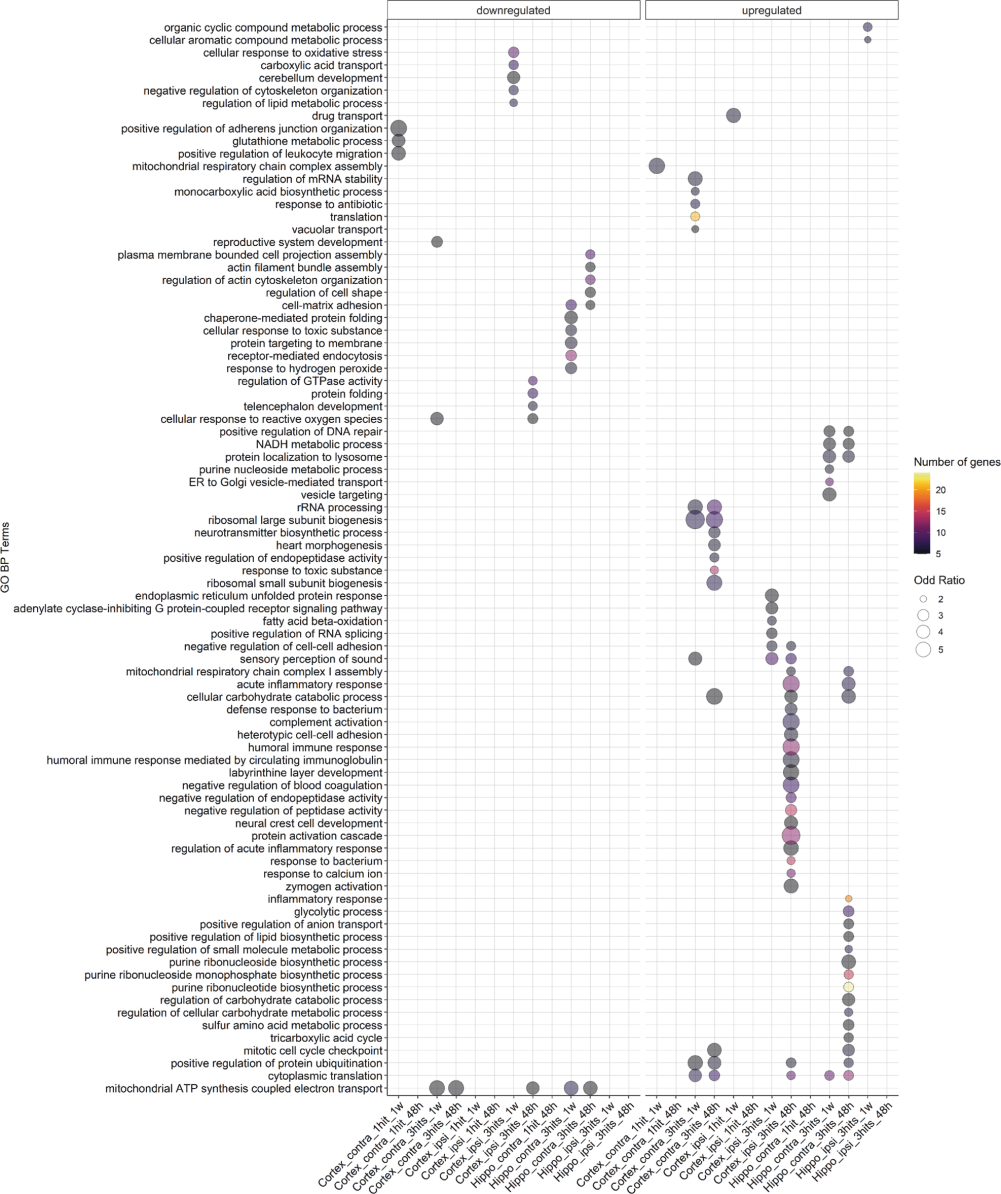
Bubble plot showing enriched/depleted GO biological process (BP) terms for various comparisons. Columns represent conditions for which GO terms are enriched for DEPs that are either upregulated (right side) or downregulated (left side) compared to SHAM. Bubble size and color reflect odd-ration and gene number of genes, respectively. Terms with an adjusted p-value lower than 0.05 in at least one condition are displayed. Hippo: hippocampus; ipsi: ipsilateral; contra: contralateral. More details can be found in Supplementary Table 4

GO enrichment analysis revealed high variability in the biological processes among different tissues and hit time points compared to the sham. The upregulated proteins were significantly enriched in 60 biological processes with the top 5 associated with ribosomal large subunit biogenesis, protein activation, complement activation, and acute inflammation. However, for downregulated proteins, 24 biological processes were enriched with the top 5 associated with adherens junction organization, mitochondrial ATP synthesis coupled electron transport, leukocyte migration, glutathione metabolic process, and chaperone-mediated folding. In summary, enrichment patterns showed high variability across all conditions with few common pathways (Fig. 3 and Supplementary Table 4).

### Biological processes were highly enriched 48 h post-injury and mostly associated with inflammatory responses

**Fig. 4:**
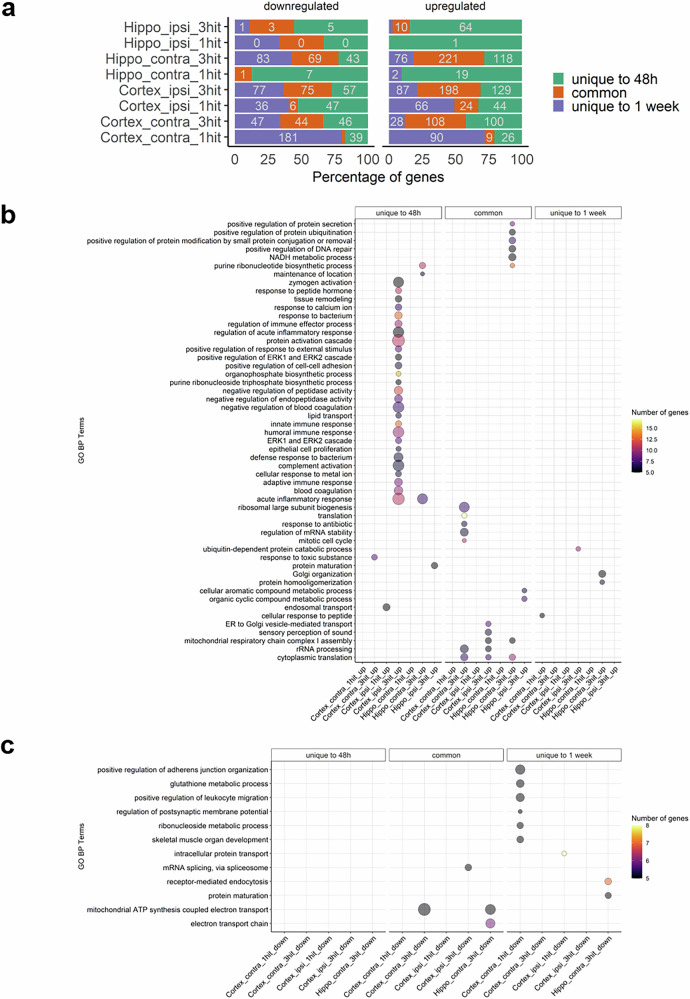
Analysis of differentially expressed proteins (DEPs) overlaps across time. **a** Barplot showing the intersections between 48 h and 1 week time points for the different hit conditions across tissues. For each condition: green color represents genes that are uniquely deregulated at 48 h compared to SHAM; purple color represents genes that are uniquely deregulated at 1 week compared to SHAM; while orange corresponds to genes that are shared between 48 h and 1 week time points. **b**, **c** Bubble plots showing enriched GO biological process (BP) terms for various conditions organized by time. Columns represent conditions for which GO terms are either enriched for DEPs that are unique to 48 h (left), 1 week (right) or shared between both time points (middle). Bubble size and color reflect odd-ration and gene number of genes, respectively. Only the terms with an adjusted p-value less than 0.05 in at least one condition are displayed. Hippo: hippocampus; ipsi: ipsilateral; contra: contralateral. More details can be found in Supplementary Tables 5 and 6

The DEPs overlap analyzed at 48 h and 1 week for the different hit conditions in the hippocampus and cortex showed 745 uniquely DEPs identified 48 h post-injury (501 upregulated and 244 downregulated) and 774 DEPs uniquely reported 1-week post-injury (349 upregulated DEPs and 425 downregulated) compared to sham (Fig. 4a) though some proteins are shared between tissues. Commonly deregulated proteins at 48 h and 1 week following injury are shown in Supplementary Table 5. GO biological process analysis demonstrated 32 biological processes upregulated 48 h after injury with the top 5 linked to protein activation cascade, acute inflammatory response, complement activation, humoral immune response, and negative regulation of blood coagulation (Fig. 4b and Supplementary Table 6). Most of these are unique to individual conditions, indicating tissue- and hit frequency-specific responses.

The only 4 upregulated processes uniquely identified 1-week after injury were correlated with ubiquitin-dependent protein catabolic processes, Golgi organization, protein maturation, and protein homo-oligomerization (Fig. 4b and Supplementary Table 6). On the other hand, 9 downregulated biological processes were exclusively enriched 1-week post-injury (Fig. 4c and Supplementary Table 6).

The commonly deregulated processes between 48 h and 1 week demonstrated 18 upregulated biological processes with the top 5 correlated with ribosomal large subunit biogenesis, rRNA processing, regulation of mRNA stability, cytoplasmic translation, and NADH metabolic process. Only 3 enriched processes were commonly downregulated and relevant to the electron transport chain, mitochondrial ATP synthesis via electron transport, as well as mRNA splicing (Fig. 4c and Supplementary Table 6). Overall, more biological processes were enriched at 48 h than at 1 week post-injury in different hit conditions and tissues.

### Biological processes were highly enriched following repetitive injury and mostly associated with cellular and inflammatory responses

**Fig. 5:**
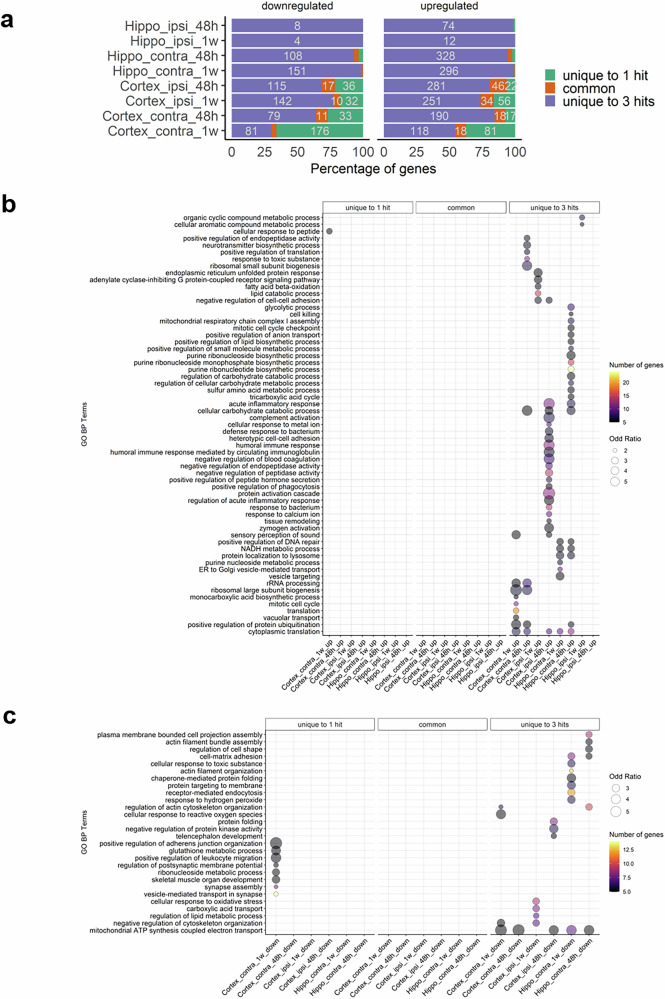
Analysis of differentially-expressed proteins (DEPs) overlap between hit conditions at specific time points. **a** Barplot showing the intersections between 1-hit and 3-hits conditions for 48 h and 1 week time-points across tissues. For each condition: green color represents genes that are uniquely deregulated at 48 h compared to SHAM; purple color represents genes that are uniquely deregulated at 1 week compared to SHAM; while orange corresponds to genes that are shared between 48 h and 1 week time points. **b**, **c** Bubble plots showing enriched GO biological process (BP) terms for various conditions organized by hit status. Columns represent conditions for which GO terms are either enriched for DEPs that are unique to 1-hit (left), 3-hits (right) or shared between both groups (middle). Bubble size and color reflect odd-ration and gene number of genes, respectively. Only terms displaying adjusted *p* value less than 0.05 in at least one condition are demonstrated. Hippo: hippocampus; ipsi: ipsilateral; contra: contralateral. More details can be found in supplementary Tables 7, 8, and 9

At 48 h and 1 week, 467 DEPs were identified at 1 hit (186 upregulated and 281 downregulated) whereas 2238 DEPs were obtained at 3 hits condition (1550 upregulated and 688 downregulated) compared to sham. 181 DEPs were common between 1 hit and 3 hits (128 upregulated and 53 downregulated) (Fig. 5a and Supplementary Table 7). Enriched GO analysis at 1 hit and 3 hits across all conditions showed distinct enrichments. Upon 1 hit, upregulated proteins were enriched in a single process implicated in the cellular response to peptides—specific to one condition. In contrast, 3 hits showed were 60 enriched biological with the top including protein activation, ribosomal biogenesis, inflammation, and immune response; several of these were shared across two or more conditions (Fig. 5b and Supplementary Table 8). Contrarily, downregulated proteins were enriched in 8 biological processes uniquely after 1 hit, including junction organization, leukocyte migration, and glutathione metabolism—all specific to one condition. After 3 hits, downregulated proteins were enriched in 20 biological processes, including mitochondrial ATP synthesis, cellular response to reactive oxygen species, and protein folding (Fig. 5c and Supplementary Table 9). No common pathways were shared between 1 and 3 hits across different tissues and time points.

**Fig. 6:**
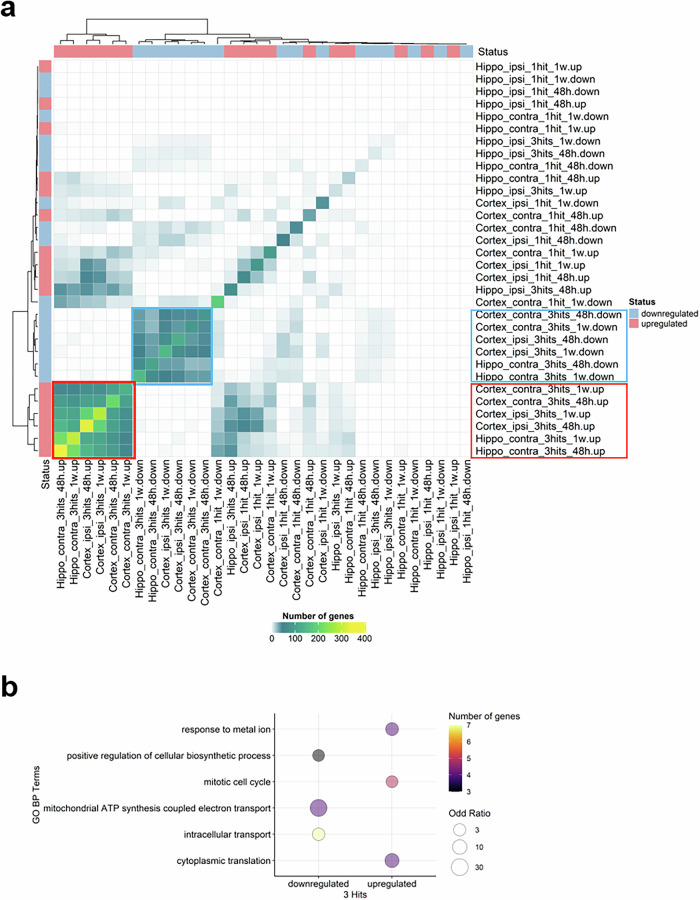
Identification of common patterns of DEPs across conditions. **a** heatmap showing all possible overlaps between up- and downregulated gene set across all conditions compared to SHAM. Rows and columns are annotated in blue and red colors to indicate up- and downregulation, respectively. Heatmap color reflects the number of overlapped genes, ranging from white (no overlap) to green and yellow, reflecting the increasing number of genes. Two clusters of constantly up- and downregulated genes across 3 tissues are highlighted in red and blue, respectively. Hippo: hippocampus; ipsi: ipsilateral; contra: contralateral. **b** Bubble plot showing enriched GO biological process (BP) terms enriched in DEPs shared across comparisons in the up- and downregulated clusters. Bubble size and color reflect odd-ratio and gene number of genes, respectively. The terms with an adjusted *p* < 0.05 in at least one condition are displayed. More details can be found in Supplementary Table 9

Moreover, an intersection analysis was established between up- and down-regulated proteins to identify the overlaps and common patterns of DEPs across all conditions compared to sham. Two clusters are identified. The data highlight two clusters of consistently deregulated proteins displaying strong overlaps (darker colors closer to yellow) with similar up- and down-regulation patterns upon 3 hits across the hippocampus contralateral, cortex (ipsi- and contralateral regions) at different time points (Fig. 6a). The identified clusters are characterized by upregulated proteins associated with cytoplasmic translation (Drg1, Rpl18, Rpl38, Rpl6), response to metal ion (Abat, Hpca, Pdcd6, Ppp2ca), and mitotic cell cycle (Drg1, Top2b, Ttyh1, Tuba1a, Tuba1b). Other enriched pathways showed downregulated proteins implicated in mitochondrial ATP synthesis coupled electron transport (Cox5a, Cox5b, Ndufs6, Snca), intracellular transport (Cox5b, Cplx2, Fabp3, Scg5, Snca, Sod1, Timm9), and positive regulation of cellular biosynthetic process (Fabp3, Snca, Tmsb4x) (Fig. 6b and Supplementary Table 9). Together, repetitive injury is characterized by more deregulated proteins and more aggravated inflammatory, energy production-linked and cellular responses.

### WGCNA analysis: co-expression network analysis uncovers hit-specific protein network alteration

To gain insights into the brain proteome changes in single and repetitive injuries across different time points, we performed WGCNA analysis which uses the whole dataset instead of being limited to the top deregulated proteins to identify a network of highly interconnected modules of co-expressed proteins.

**Fig. 7:**
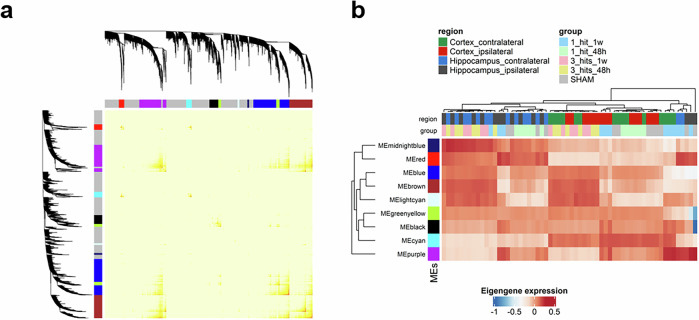
Weighted gene co-expression network analysis (WGCNA). **a** Topology overlap matrix displaying 10 modules that are shown with distinct colors. The gray color corresponds to a background module containing all proteins which were not associated with any other module. **b** Heatmap illustrating the module eigengenes (MEs) presenting the 9 modules of interconnected proteins. For every module, the eigengene corresponds to the first principal component (PC1) obtained from the PCA performed on the expression matrix of the associated genes. Hierarchical clustering for rows and columns was done using Euclidean distance with average linkage. Columns are annotated by tissues and experimental groups. Heatmap color is proportional to eigengene value (More details can be found in Supplementary Table 10)

WGCNA identified nine modules of strongly connected proteins each assigned a color, and ranging in size from the largest (blue module: Data preparation and quality control steps are detailed in Supplementary Fig. S5 570 proteins) to the smallest (lightcyan module: 31 proteins) (Fig. 7a). The gray module represents proteins which failed to group into a defined module. Each module is characterized by an eigengene, a representative synthetic gene, defined as the first principal component (PC1) that explains the highest variance in expression patterns among all proteins within that module. Hierarchical clustering analysis based on the module eigengenes indicated hit-specific correlation associated with several module eigengenes (MEs) (Fig. 7b). For instance, the brown (denoted by MEbrown) and the light cyan (denoted by MEcyan) modules were highly expressed with 3 hits compared to sham, whereas the expression of the purple module (MEpurple) decreases with 3 hits compared to 1 hit in all tissues and independently of the different time points. Therefore, several modules were correlated with specific hit conditions, indicating that the primary variation across the dataset is attributed to the difference in hit conditions (Fig. 7b and Supplementary Table 10).

**Fig. 8:**
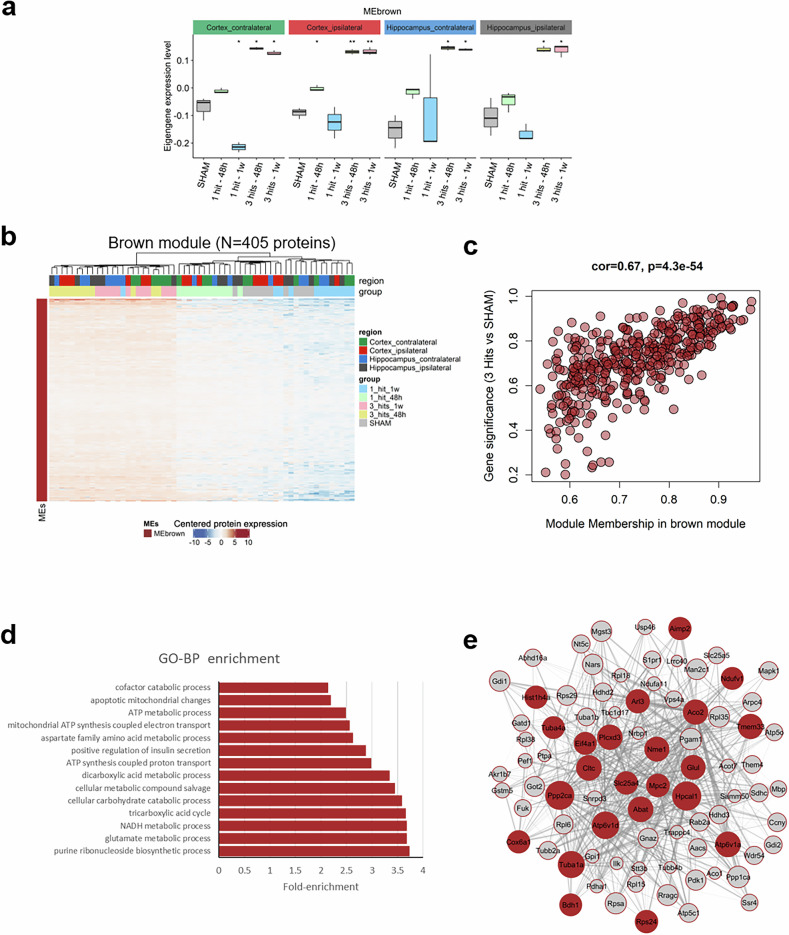
Analysis of the brown module. **a** Boxplot demonstrating the changes in the brown module eigengene (MEbrown) across the SHAM, with the various Hit-Time conditions for each tissue. Student’s *t*-test was used to compare various conditions to SHAM. (*: *p* < 0.05; **: *p* < 0.01). **b** Heatmap showing the 405 proteins expression profile found in the brown module. **c** Scatter plot showing the correlation between Gene significance (GS) and Module membership. GS was described as the correlation of protein expression with 3-hits vs SHAM, regardless of tissue of origin. Module Membership (MM) corresponds to the correlation between protein expression and the module eigengene. The higher the GS score, the more a protein is increased by 3-hits compared to SHAM. The higher the MM score, the more a protein is interconnected within the module. A high positive correlation between GS and MM (*r* = 0.67, *p* = 4.3 × 10^−54^) demonstrates that the brown module constitutes an interconnected network of proteins that is increased by 3-hits. **d** GO biological process enrichment results for proteins found in the brown module. More details about enriched terms can be seen in Supplementary Fig. 6. **e** protein-protein interaction (PPI) network showing the top interconnected proteins within the brown module (weighted correlation > 0.1). The Node size and the edge width correspond to module membership (MM) and weighted correlation, respectively (the top 25% edges are only visualized). Hub proteins with an MM score above 0.9 are colored in brown. More details about brown-module proteins can be found in Supplementary Table 10

The global expression of the MEbrown shows an increase in the expression of the eigengene specifically with 3 hits compared in all tissues compared to the shams (Fig. 8a). The identified 405 proteins in the MEbrown displayed higher expression in the 3 hits samples across all tissues, which clustered separately from the sham and 1 hit groups (Fig. 8b and Supplementary Table 10). Analysis and revealed a strong positive correlation with of the module membership (MM, the correlation between a protein’s expression and the module eigengene) and gene significance (GS, the correlation between a protein’s expression and a 3-hits compared to sham) revealed a strong positive correlation the higher expression with the 3-hits compared to the sham (*r* = 0.67, *p* = 4.3 × 10^−54^; Fig. 8c and Supplementary Table 10). This demonstrates that the brown module constitutes a highly interconnected network of proteins that are increased by 3-hits regardless of tissue of origin and time- points conditions (Fig. 8c and Supplementary Table 10). Interestingly, the GO analysis indicates that the brown module proteins are majorly associated with mitochondrial activity and metabolic processes (Atp6v1a, cyc1, Cox6a1, Mapk9, Ndufs1/2, Ndufv1, Slc25a4, Glul, Got1, Eno2) (Fig. 8d and Supplementary Fig. 6). The protein-protein interaction PPI network mapping of the hub proteins within the brown module identified a top of 23 highly interconnected proteins (*colored in brown*; Fig. 8e and Supplementary Table 10) of which 9 metabolic proteins were critical and correlated with metabolic and mitochondrial functioning (Ndufv1, Aco2, Nme, Glul, Mpc2, Slc25a4, Abat, Cox6a1, and Atp6v1).

The light cyan module exhibits increased expression (31 proteins) with 3 hits compared to sham, like the brown module, and the proteins are majorly implicated in tissue development (Supplementary Fig. 7). To note, the light cyan module tends to increase with 1 hit at 1 week whereas the brown module is decreased. This implies that the brown module pathways are 3-hit specific while the light cyan pathways tend to be *latent post-1-hit traumas*.

**Fig. 9:**
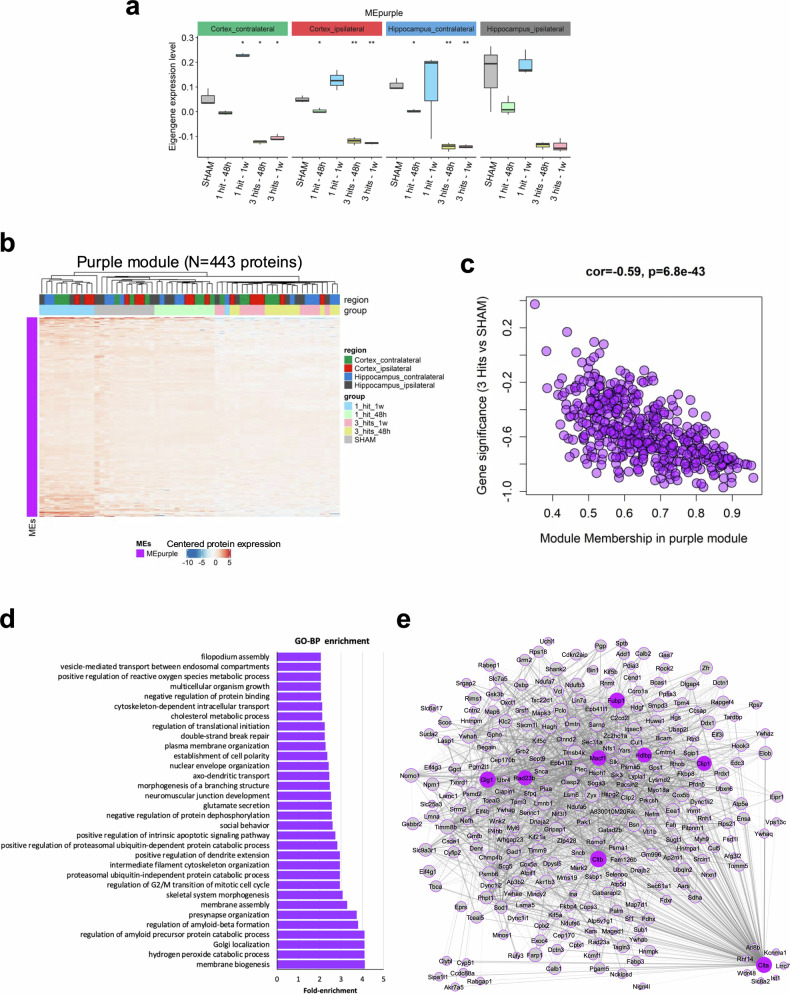
Analysis of the purple module. **a** Boxplot demonstrating the changes in the purple module eigengene (MEpurple) across the SHAM and various Hit-Time conditions for each tissue. Student’s *t*-test was used to compare various conditions to SHAM. (*: *p* < 0.05; **: *p* < 0.01). **b** Heatmap showing the expression profile of the 443 proteins found in the purple module. **c** Scatter plot showing the correlation between the Gene significance (GS) and the Module membership. GS was described as the correlation of protein expression with 3-hits vs SHAM, regardless of tissue of origin. Module Membership (MM) represents the correlation between the expression of the protein and the module eigengene. The lower the GS score, the more a protein is decreased by 3-hits compared to SHAM. The higher the MM score, the more a protein is interconnected within the module. High negative correlation between GS and MM (*r* = −0.59, *p* = 6.8 × 10^−43^) demonstrates that the purple module constitutes an interconnected network of proteins that is decreased by 3-hits. **d** GO biological process enrichment results for proteins found in the purple module. More details about enriched terms can be seen in Supplementary Fig. 8. **e** protein-protein interaction (PPI) network showing the top interconnected proteins in the purple module (weighted correlation > 0.1). The Node size and the edge width correspond to module membership (MM) and weighted correlation, respectively (only the top 25% edges are visualized). Hub proteins with an MM score above 0.9 are colored in purple. More details about purple-module proteins can be found in Supplementary Table 10

In contrast to the brown and light cyan modules, the MEpurple showed decreased expression with 3 hits compared to sham across different time points regardless of the tissue origin at different time points (Fig. 9a). This is confirmed by the decreased expression of MEpurple’s 443 proteins in 3 hits, which clustered separately from 1 hits and sham groups (Fig. 9b and Supplementary Table 10**)**. A high negative correlation between the module membership (MM) and gene significance (GS) is observed (*r* = -0.59, *p* = 6.8 × 10^−43^; Fig. 9c and Supplementary Table 10) illustrating that the purple module constitutes an interconnected network of proteins that is decreased by 3-hits. The purple module proteins are involved in cytoskeletal organization, morphogenesis, pre-synapse organization, and other neuron-related terms (Shank1, Kif5a, Map6, Sod1, Snca, Ensa) (Fig. 9d and Supplementary Fig. 8). The protein interaction network analysis examining the hub proteins within the purple module displayed a top of 8 highly interconnected proteins such as Cltb, Glg1, Macf1, Fubp1, and Clip1 (highlighted in purple, Fig. 9e and Supplementary Table 10).

Overall, these findings highlight proteomic alterations across brain regions that are exclusive to repetitive trauma and provide candidates for further functional investigation.

### Single and repetitive mTBI and rmTBI impair the gross motor function at the acute and sub-acute Stages

**Fig. 10:**
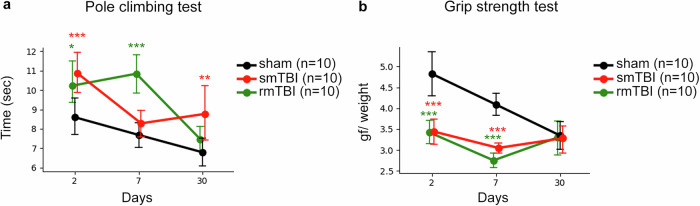
Assessment of motor function deficits. The pole climbing test for motor coordination (**a**) and the grip strength test for muscle strength (**b**) were conducted to assess gross motor function. The plots display mean climbing time (±CI) across 3 timepoints 2, 7, and 30 days after injury: Sham (black, *n* = 10), smTBI (red, *n* = 10), and rmTBI (green, *n* = 10). At day 2 post-injury (**a**), the smTBI (***p < 0.001) and rmTBI (*p < 0.05) groups took more time to descend the pole in comparison to sham, whereas at day 7 the rmTBI group took more time than sham (***p < 0.001) to descend, indicating more severe motor coordination deficits. At 30 days following injury, smTBI groups displayed mild impairments compared to the sham (**p < 0.01). In the grip strength test (**b**), the sham group performed better than both smTBI (***p < 0.001) and rmTBI (***p < 0.001) at days 2 and 7 post-injury. No significant differences were observed at day 30 in either test (*p* > 0.05). Statistical comparisons by linear mixed-effects models (LMM). Error bars (±95% CI) reflect the uncertainty of the mean estimate at each time point

The gross motor performance was assessed in mice with single (1 hit) and repetitive (3 hits) mTBI at day 2 (acute), 7 (sub-acute), and 30 (chronic) post-TBI via pole climbing and grip strength tests (Supplementary Fig. 1 and Supplementary Table 11). In the pole climbing test on day 2 post-TBI, both mice with smTBI and rmTBI needed more time to descend the pole in comparison to the sham group (*p* = 0.001, *p* < 0.05, respectively), suggesting early disrupted motor coordination (Fig. 10a). At day 7 post-injury, mice with rmTBI significantly took more time to descend in contrast to the sham group (*p* < 0.001) revealing more severe motor balance and coordination deficits. At 30 days following injury, smTBI groups displayed significant mild impairments by taking more time to descend the pole compared to the sham (*p* < 0.01), unlike the rmTBI group that showed improved performance similar to the sham group. Muscle strength was significantly decreased in both smTBI and rmTBI groups at 2- and 7-days post-injury compared to the sham (both *p* < 0.001) (Fig. 10b). In both injured groups, no significant changes in motor coordination and muscle strength were observed at day 30 post-TBI compared to the sham, which may indicate recovery. These results demonstrate that single or repetitive mTBI impaired the gross motor function at acute or sub-acute injury Stages.

### Repetitive mTBI displays learning deficits at the sub-acute and chronic Stages

**Fig. 11:**
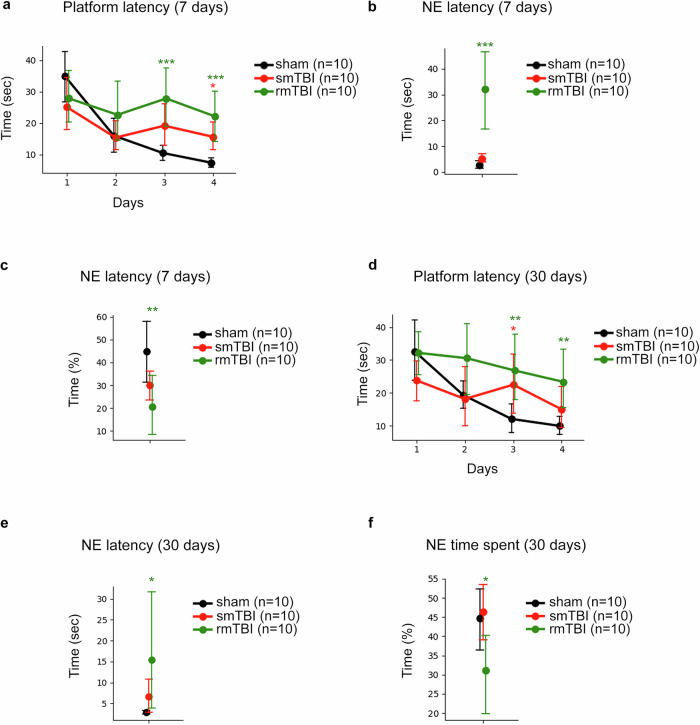
Spatial learning and memory at subacute (7 days) and chronic (30 days) stages. Sham (black, *n* = 10), smTBI (red, *n* = 10), and rmTBI (green, *n* = 10). **a** Acquisition latencies in the Morris water maze in the sub-acute (day 7) Stage group; rmTBI mice took the longest time, significantly on acquisition days 3 (***p = 0.001) and 4 (***p < 0.001); smTBI group showed significant deficits on the fourth acquisition day (*p < 0.05) com*p*ared to the sham. **b** Probe trial NE quadrant latency (subacute); rmTBI required more time (***p < 0.001) than sham. **c** Probe trial % time in NE quadrant (subacute); rmTBI spent less time (**p < 0.01) than sham. **d**–**f** Chronic stage (day 30) analogs: (d) acquisition day 3 showed smTBI > sham (*p* < *0.05) and rmTBI > sham (**p < 0.01); acquisition day 4 showed rmTBI > sham (**p < 0.01); **e** probe latency showed rmTBI > sham (*p < 0.05), and (**f**) % time where rmTBI < sham (*p < 0.05), and no smTBI deficits were observed. Statistical comparisons by linear mixed-effects models (LMM). Error bars (±95% CI)

Cognitive impairments induced by TBI were investigated using the MWM test on mice subjected to smTBI and rmTBI and sacrificed 7- and 30-days after injury (Fig. 11 and Supplementary Table 11). The latency to find the hidden platform was evaluated. In the sub-acute (day 7) Stage group, the rmTBI mice displayed significant impairments on the third (*p* = 0.001) and fourth (*p* < 0.001) acquisition days compared to the sham, whereas the smTBI group showed significant deficits on the fourth acquisition day (*p* < 0.05) compared to the sham (Fig. 11a). During the probe trial day, there was a significant impact on the latency period noticed by the rmTBI mice that required additional time (*p* < 0.001) to find the target NE quadrant (Fig. 11b) while spending less percentage of time in the NE quadrant (*p* < 0.01) in contrast to the sham group (Fig. 11c). This suggests that the rmTBI group exhibits deficits in learning and memory retention 7 days post-injury. In the chronic Stage group (day 30 post-injury), the latency to reach the platform was significantly longer in the smTBI and rmTBI mice on the third acquisition day (*p* < 0.05, *p* < 0.01, respectively) and only in the rmTBI mice on the fourth acquisition day (*p* < 0.01) compared to the sham (Fig. 11d). On the probe day, the rmTBI needed more time to locate the target NE quadrant (*p* < 0.05) (Fig. 11e) while spending less percentage of time in that quadrant (*p* < 0.05) compared to the sham animals (Fig. 11f). These observations imply that repetitive mTBI results in significant severe deficits in spatial memory and learning performances in mice 30 days post-injury. The smTBI showed mild deficits in the latency to find the target quadrant but in the time spent in that quadrant in sub-acute and chronic Stages, which indicates that our single mTBI model may induce mild memory retention deficits. Our findings imply that repetitive mTBI impairs cognitive memory and learning performance in mice 7 or 30 days following the injury.

### Sham and mTBI mice display anxiety-like behavior at the sub-acute and chronic Stages

**Fig. 12:**
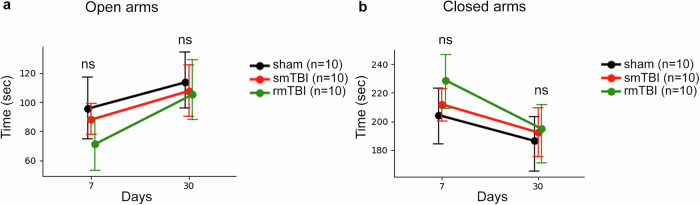
Assessment of anxiety-like behavior at the subacute and chronic time points. Anxiety-like behavior at subacute (7 days) and chronic (30 days) stages. Time spent in open (**a**) and closed (**b**) arms of the elevated plus maze. Sham (black, *n* = 10), smTBI (red, *n* = 10), and rmTBI (green, *n* = 10). All groups spent similar time, indicating no group differences in anxiety-like behavior. Statistical comparisons by linear mixed-effects models (LMM). Error bars (±95% CI); ns, not significant: *p* > 0.05

Anxiety-like behavior among the groups at the subacute and chronic time points was evaluated using the elevated-plus maze (EPM) that examines the mice preference for dark closed arms of the maze over bright open arms under stressful conditions (Fig. 12 and Supplementary Table 11). No significant differences were noticed between sham and injured mice (smTBI and rmTBI) within the same open-arms (Fig. 12a) or closed-arms groups (Fig. 12b) at sub-acute or chronic time points. These findings suggest that the observed anxiety-like behavior is not time-dependent and not correlated with the single or repetitive mTBI model. This outcome eliminates the possibility of anxiety parameter affecting the mice performance in other tests.

## Discussion

This study provides a detailed proteomic analysis of single and repetitive mild traumatic brain injury (mTBI) in mice, focusing on early (48-h) and subacute (1-week) time points. By exploring injury-related changes in the cortex and hippocampus regions critical for cognitive and motor function, we have identified specific injury-driven proteomic alterations. These findings highlight molecular pathways potentially contributing to the heightened risk for neurodegeneration in repetitive mTBI and delineate associated behavioral deficits. Our analysis demonstrates that both single and repetitive mTBI provoke distinct and region-specific alterations in protein expression within the cortex and hippocampus, with repetitive TBI resulting in more severe disruptions. Consistent with previous studies on TBI-induced cellular stress and mitochondrial dysfunction our data indicate that repetitive mTBI exacerbates dysregulation of proteins involved in mitochondrial respiration and synaptic plasticity (e.g., Cox6a1, Snca).^36–39^ This suggests that repetitive injury may intensify vulnerability to neurodegenerative processes observed in conditions like AD and PD, which are often marked by similar proteinopathies.^40–43^

Proteomic profile analyses of a single and repeated mild TBI mouse model at 48 h and 1 week, delineate the acute and subacute Stages of clinical TBI. We aimed to identify differentially expressed proteins and the corresponding biological processes affected in the ipsilateral and contralateral regions of the cortex and hippocampus. The identified deregulated proteins were analyzed by GO enrichment, WGCNA, and PPI functions. Our proteomic analysis between all experimental groups in the hippocampus and cortex allowed us to identify several Stages of biological processes within the acute and subacute injury Stages in single and repetitive TBI, where some Stages were injury-related in terms of biological processes involved. In the single hit, several common Stages across different tissues were implicated in the exacerbation of axonal injury in the acute Stage (48 h post-injury) and possible recovery in the subacute Stage (1-week). In the ipsilateral hippocampal region, Stage 1 was implicated in neurotransmission (Rims1) and stress-related response (HSPA1L), which were upregulated 48 h post-injury as possible protection of the proteome from stress, then restored to control levels 1-week after TBI. Similarly, in Stage 4, the expression of proteins involved in maintaining cell survival (Ptrh2) was restored 1 week after TBI. Other proteins were involved in inflammation as in Stage 3 (C1qa), which is in line with our previous study with a TBI rat model.^14^ Complement is known to drive synaptic degeneration and progressive cognitive decline in the Chronic Stage after Traumatic Brain Injury.^44^ Consistent with this, Stage 6 in the ipsilateral cortex, represented by a large group of proteins, showed a restoration of several neuroprotective biological pathways in the subacute injury Stage (1 week). These include several heat shock-related proteins (Hspa2, Hspa5, Hspa9), FABP7, and Basp1, which are correlated with axonal regeneration and myelination, in addition to protective antioxidative stress regulators (Prdx1, Gpx1). Other proteins have been implicated in the regulation of proper synaptic neurotransmission, such as Nrxn1. Interestingly, in the contralateral hippocampus, some proteins associated with learning and synaptic transmission such as neuronal pentraxin receptor (Nptxr) and synaptotagmin 11 (Syt11) were downregulated in the late subacute injury Stage. In this context, neuronal pentraxins have been reported to be involved in neurodegeneration and dementia. Furthermore, deletion of Syt11 correlated with impaired memory and synaptic plasticity. The obtained results are consistent with the ones, we performed on an experimental TBI rat model of moderate controlled cortical impact injury using spatially resolved microproteomics to assess the protein profile at four injury time points (1 day, 3 days, 7 days, and 10 days) depicting the acute, subacute and subchronic Stages of clinical TBI.^14,23^ Four Stages of biological processes occur within the first 10 days post-impact. Although Stage 1 (a and b) was characterized by proteomic pathways that are usually depicted in uninjured conditions, Stages 2, 3, and 4 explained better the injury-related processes in a time course. Stage 2 was identified by proteins associated with acute inflammation and infiltration of blood-related proteins linked to a breach within the BBB. Surprisingly, Stage 3, which corresponds to the proteins elevated at 3 and 7 days postimpact, showed a dual effect regarding survival and death of cells in a synchronized manner. Although the dying cells undergo programmed cell death execution such as apoptosis, the surviving cells located in the same spatial distribution trigger DNA repair mechanisms by upregulating the expression of multiple ribosomal proteins associated with the translation process. Therefore, DNA repair is activated as early as 3 days following the impact in TBI. Furthermore, the brain’s proteomic profile tends to recover to a profile similar to the sham, as we have demonstrated in this study.^14^ These outcomes highlight the remarkable plasticity factor of the brain, with its capacity to attempt to restore a normal protein profile even 10 days after the mechanical impact. We recently demonstrated that among the neural cells involved, microglial cells may trigger the early stage of the disease through extracellular vesicles containing acylcarnitines production at the injury site which impacts the microglia in *substantia nigra* driving the propagation of inflammation.^13,45^ Moreover, acylcarnitines also act towards astrocytes and promote regeneration and axonogenesis.^23^ We recently demonstrated that upon inflammation, astrocytes produce aberrant immunoglobulins which bind to neurons through Fc receptor and activate neurogenesis processes^46,47^ and can participate in this repair process in smTBI.^48,49^

On the other hand, proteomic Stages identified in rmTBI were mostly correlated with aggravated axonal injury and cell death, with a few Stages correlated with potential neuronal recovery.^50^ In the ipsilateral hippocampus, Stage 1 showed downregulation in leukocyte migration, neurotransmission, and responses to free radicals at both acute and subacute time points. Similarly, additional Stages involved in axonal regeneration were downregulated in the contralateral hippocampus. Another major set of proteins was correlated with increased neuronal damage in the ipsilateral cortical region (Stage 4). Several axon guidance and neurogenesis-related pathways were downregulated along with neuroprotective antioxidant processes. For example, ephrin type-A receptor 4 (Epha4), which plays an essential role in axon guidance, is also implicated in synaptic plasticity and learning. Other axon guidance signaling-related proteins in our proteome were also downregulated, suggesting failed recovery at the subacute time point following repetitive concussion. Our findings align with a previous study, which reported a downregulation of axon guidance signaling-related proteins in postmortem chronic traumatic encephalopathy brain.^51,52^ These results suggest that repair-associated mechanisms were functionally impaired after repetitive mTBI in contrast to single mTBI. To our attention, a small group of proteins involved in angiogenesis, infiltration of blood-related proteins, and acute inflammation were restored to the sham condition, suggesting a possible BBB breach in the acute injury Stage documented as well in our previous study and others, which is restored here in the subacute Stage after repetitive injury.^14,53–55^ Vascular damage plays a critical role in the development of secondary injury in response to TBI by disrupting the BBB and disrupting blood flow, and it has been proposed that vascular repair is initiated 2–3 weeks after injury. In addition, upregulation of cellular respiratory pathways was observed at multiple stages, as indicated by stages 3 and 4 in the ipsilateral cortex and hippocampus, respectively. Remarkably, a dual effect in terms of cell death and survival was observed in Stage 4 (ipsilateral hippocampus), with proteins highest at baseline (sham) that drop after injury and stay low at 48 h and 1 week post-injury. In this Stage, surviving cells initiated DNA repair by upregulating ribosomal proteins involved in translation, while dying cells underwent stress and programmed cell death executed by proapoptotic proteins. Interestingly, at the same time, several chromatin remodeling proteins associated with transcriptional repression were upregulated, such as histone deacetylase 11 (Hdac11). It has been reported that the upregulation of Hdac in the hippocampus affects learning and memory in the repetitive mTBI rat model. The inhibition of Hdac has been documented to alleviate neuroinflammation and improve cognitive performance in rodent TBI models.^56^ Following rodent TBI, several cognitive functions such as spatial and fear learning are reported to be enhanced by HDAC inhibitors.^57,58^ The data imply that suppression of HDAC could reduce neuroinflammation, which strongly contributes to the long-term connection between TBI and the later development of NDs.

Accordingly, repair-associated mechanisms displayed 1-week post-injury imply that the proteomic profile of the brain has the potential to be restored to the sham condition with a single and not repetitive impact. This finding suggests an appropriate neural plasticity possessed by the brain to recover the normal proteomic profile after the subacute Stage of a single mechanical injury with a dominant neurological decline manifested in the subacute Stage, in contrast to the acute Stage with repetitive injury. This aligns with our previous outcomes with the TBI spatiotemporal proteomics analysis where the brain protein profile was restored to the non-injured condition at the subacute Stage after injury.^23^

Furthermore, GO analysis performed in the injured versus sham groups indicated that the acute Stage was enriched in inflammation-related pathways in accordance with previous multiplexed proteomics results in a weight-drop mTBI mouse model^23^ and others, whereas repetitive TBI showed pathways enriched in cellular and inflammatory responses, similarly to a former proteomic study reporting persisting inflammatory pathways at acute and chronic time-points in repetitive mTBI.^53^ Consistent with the previous data, the WGCNA analysis suggests increased mitochondrial metabolic processes with repetitive, rather than single, mechanical injury, represented by increased cellular activity and energy production, and decreased neural-related pathways, including neurogenesis and synaptic transmission. It has been documented that a complex of neuroprotective and reparative responses is initiated by injury, but prolonged activation of these mechanisms may attenuate the progression of neurodegeneration, apoptosis, and long-term neuroinflammation.^59^ Our proteomic analysis revealed the deregulation of several proteins, some of which are associated with neurodegenerative disorders. Indeed, mild TBI, and especially repetitive TBI, is recognized as a risk factor for neurodegenerative diseases, including AD and PD, and others.^60,61^ In addition, the risk of long-term dementia has been documented to increase after TBI, suggesting a potential association with dementia-related ND. Among the deregulated proteins identified in our study, the mitochondrial-related Cox6a1 has been reported to be involved in PD, where mitochondrial dysfunction has been proposed as a pathological driver of the early stages of PD and AD. Nrgn is a post-synaptic protein involved in visual and spatial learning and memory.^62^ In AD, Nrgn, which is involved in synaptic transmission, has been selectively associated with tau and amyloid pathology in postmortem cortical brain regions.^62^ In addition, ApoE, a precursor of ß-amyloid involved in lipid metabolism, has been implicated in the exacerbation of neurodegeneration after TBI.^63^ In AD, ApoE has been correlated with the clearance of synaptic amyloid beta peptide, with elevated levels of ApoE documented in the synaptic terminals of AD’s rat models and patients.^64^ Additional evidence has highlighted a toxic role of ApoE in exacerbating tau-mediated neurodegeneration, which adversely affects cognitive function in patients with acute mTBI.^65^ Another protein involved in lipid metabolism is Apoa1, which was upregulated after injury in this study and may be associated with neurodegeneration in patients with MS, AD and PD.^66^ In MS, decreased levels of Apoa1 have been reported to correlate with neuroaxonal injury in patients, which may adversely affect learning and memory, as several studies have documented a protective role of elevated Apoa1 levels in delaying the progression of cognitive deficits.^67–69^ In addition, serum levels of Apoa1 have been reported to be downregulated in patients with AD, suggesting that Apoa1 deficiency may be a potential risk factor for AD. Moreover, Apoa1 is the most thoroughly studied marker in PD among the apolipoproteins, where low levels of Apoa1 have been associated with the vulnerability of the dopaminergic system in symptomatic patients, suggesting its contribution to the early onset of PD.^70,71^ Recently, the pathogenic role of the ribosomal-related protein Rpl35 has been investigated in the AD mouse model. It has been suggested that Rpl35 may contribute to the development of AD by disrupting cellular processes and producing toxic aggregates after initiating protein misfolding.^70^ Our proteomic data showed deregulation in several NADH dehydrogenase (ubiquinone) subunits such as Ndufa3 and Ndufb3, which were correlated with several NDs including AD and HD. In a TBI rat model, Ndufa3, Ndufb3, and other subunits were identified among the genes correlated with neurodegenerative diseases. Notably, Gad1 was overexpressed among the contralateral deregulated proteins in the cortex, although it was not captured among the top 20 overlapping proteins with ND. Notably, Gad1 was characterized as one of the signature protein markers in the substantia nigra of a rat TBI model that correlates with PD.^23^

Finally, several dementia-related proteins were deregulated after injury in our study. These include glutamate ionotropic receptor AMPA type subunit 4 (Gria4) and synuclein alpha (Snca).^72,73^ In a proteomic approach performed on postmortem human brains with AD and Parkinson’s disease with dementia (PDD), and dementia with Lewy bodies (DLB), a significant correlation was found between cognitive impairment and loss of Gria3 protein before death. Specifically, a decrease in Gria3 expression was reported in AD, whereas Gria4 was pathologically associated with PDD and DLB.^72^ Interestingly, Gria4, which has been identified as a synaptic marker of cognitive decline in neurodegenerative diseases, is downregulated in the ipsilateral cortical region in the acute injury Stage after single TBI, which may suggest a predictive molecular fingerprint of Gria4 and other synaptic proteins in our proteome associated with neurodegeneration after TBI. In addition, Snca is another dementia-related protein that was uniquely downregulated in the hippocampus with repetitive TBI.^74,75^ Snca is highly associated with neuronal activity and has been implicated in several synucleinopathies,^76^ including PD, DLB, and AD. Downregulation of Snca has been considered a potential neuroprotective approach in the final stages of neurodegeneration. It has been shown that axonal transport is disrupted in response to brain injury, which has been reported in our proteome mainly after repetitive TBI. This may lead to the accumulation of α-synuclein following neuroinflammation and; subsequently, alter behavioral outcomes. A possible neuroprotective mechanism has been proposed by reducing the accumulation of α-synuclein after mTBI. In our study, Snca was downregulated specifically in the hippocampus after repetitive TBI, which may indicate a possible neuroprotective strategy in response to the exacerbated neuronal damage and impaired neuronal repair mechanisms correlated with repeated mechanical impact. In our view, the present identified proteins associated with neurodegeneration post-TBI, may serve as candidate biomarkers to enhance the outcome prognosis following neuronal damage.^77,78^ Establishing a solid link between the injury state and the associated biomarkers accelerates the development of new treatments, while the FDA approval of neurodegeneration biomarkers may facilitate the introduction of novel treatments for TBI-induced neurodegeneration in clinical trials. The upregulated chromatin remodeling proteins in our proteome display a potential therapeutic intervention to enhance cognitive performance and neuroinflammation as previously reported with Hdac inhibitors. Several neurodegeneration protein biomarkers such as Tau and Apoa1 have been considered promising for the diagnosis of TBI.^79^ Apolipoprotein A1 which displays a promising therapeutic intervention in ALS is denoted as a biomarker for TBI that can be used in therapeutic interventions for neurodegeneration following TBI.^79–81^ Moreover, the micro^82^ tubule-associated protein tau significantly correlates with the degree of injury and may be used as a biomarker in the assessment of axonal damage post-TBI neurodegeneration^83^ {Zhang, 2025 #97}. In preclinical models, circulating blood Tau was assessed in several single and repetitive TBI studies as a potential pharmacodynamic marker. Incorporating potential biomarkers in preclinical studies may help align preclinical models to human TBI by employing the biomarker trajectories as anchor points to enhance transnational research.^82,84^

We further demonstrated that our single and repetitive injury models resulted in motor impairments in the acute and subacute injury Stages (days 2 and 7 post-impact), which were partially recovered in the chronic injury Stage (30 days post-injury). However, our rmTBI model resulted in cognitive memory and learning deficits at the subacute and chronic time points, reflecting the diffuse nature of the injury. These findings are consistent with other research studies in repetitive mTBI models.^85–87^ To our knowledge, cognitive performance in the MWM reflects hippocampal integrity, and cognitive change in response to concussion is a complex, multifactorial process. Cognitive deficits that occur in the subacute Stage may be partially explained by deficits in the synaptic neurotransmission.^88^ Our proteome reflected deregulation in several proteins associated with synaptic transmission and associative learning such as Gria4, Nptxr, Nptx2, and Snca. Therefore, the alterations in synaptic transmission are likely to be partly responsible for the cognitive deficits regulated by the hippocampus after repetitive injury. This is consistent with other studies reflecting learning and memory impairments in response to the repetitive mTBI model.

Our behavioral assays underscore the functional consequences of proteomic disruptions caused by single versus repetitive mTBI. Both injury types affected motor function and grip strength during the acute and subacute Stages, though complete recovery was observed by the chronic Stage (30 days post-injury) in the repetitive injury. In contrast, spatial learning and memory deficits were more pronounced and persistent in the repetitive mTBI group, as shown by MWM performance. The observed deficits in cognitive function align with proteomic changes in the hippocampus, a region vital for memory consolidation, specifically, proteins implicated in synaptic transmission and neuroplasticity were deregulated in the repetitive injury model, supporting the association between TBI-induced proteomic changes and cognitive impairment. Notably, anxiety-like behavior did not differ significantly between groups, ruling out general stress-related factors as contributors to the cognitive and motor deficits observed, a finding consistent with similar assessments in TBI research.^89–92^

The study was performed to capture the proteomic and behavioral changes over specific time points in control sham and injured mice. Several limitations should be noted. First, our sham mice were anesthetized with the same schedule as injured mice, but the effects of repeated anesthesia per se were not tested. In fact, including a sham-with-anesthesia control would strengthen future interpretations. The effect of repeated anesthesia exposures without brain injury in the sham group would allow for a more efficient interpretation of the proteomic/molecular changes attributed to the TBI itself. Introducing the timing differences between the TBI single and repetitive groups in future studies would help to alleviate the confounding timing impact of sample collection between the different groups. Second, although we sampled multiple time points (48 h, 1 week, and behavior up to 30 days), chronic proteomic changes beyond 30 days remain unexplored. Thus, an extended longitudinal analysis beyond 30 days would be informative to address the chronic proteomic changes and long-term neurodegenerative outcomes associated with single and repetitive TBI. Third, most importantly, the proteomic data was initially validated using different LFQ algorithms (MaxQuant 2.6.7.0 and Proteome Discoverer 2.5) to handle protein inference, normalization, and missing data differently. This validation was carried out using machine learning to impute or correct missing values. However, *some of the identified proteins exhibited* systematic bias. To address this, we reanalyzed our samples on a more powerful and advanced instrument (timsTOF fleX) employing Data independent acquisition analysis (DIA-NN). We then cross-analyzed DDA (Data-Dependent Acquisition) with Machine learning (ML) and DIA (Data-Independent Acquisition) using DIA-NN. From this cross-validation, we identified a set of specific proteins quantified as presented in Supplementary Fig. 2 as a bubble Plot and in the Supplementary Fig. 4. Western blots (Supplementary Fig. 3) were conducted as preliminary pilot experiments on independent sham, smTBI, and rmTBI samples (48 h post-injury) targeting TUBa1a, Snap25, and RAD23b. The results display trends consistent with those observed in the DDA-ML and DIA-ML analyses. It is important to note that these validations are preliminary, with comprehensive validation to be addressed in future work. While Western blots can support proteomics findings for a few proteins (particularly abundant ones with high-quality antibodies), they are not suited for validating large datasets or substituting proteomic quantification.

This study provides a comprehensive proteomic and behavioral analysis of single and repetitive mild TBI in a mouse model. Gene Ontology (GO) enrichment analysis further revealed that repetitive TBI drives early inflammatory and metabolic responses in the cortex, in line with known TBI pathophysiology. Importantly, the application of weighted gene co-expression network analysis (WGCNA) provided additional insights, uncovering networks of co-regulated proteins specifically associated with injury conditions.^93,94^ The identification of co-expression modules, particularly those related to mitochondrial dysfunction, cellular energy processes, and inflammatory response, sheds light on how repetitive injuries might lead to cumulative cellular stress and impaired neural resilience.^89,90^ Thus, our findings highlight distinct temporal and regional changes in the brain proteome that correlate with motor and cognitive deficits, particularly under conditions of repetitive injury. The identified protein networks associated with mitochondrial dysfunction, inflammatory response, and synaptic regulation contribute to the understanding of TBI-induced neurodegenerative risk. By identifying injury-specific molecular proteomic signatures, this study lays a foundation for future research into biomarkers and therapeutic targets that could mitigate long-term cognitive decline in individuals experiencing repetitive head trauma as has been discussed in previous studies.^95,96^

## Materials and methods

### Animals

All the experimental procedures involving animals were reviewed and approved by the Institutional Animal Care and Use Committees (IACUC) at the American University of Beirut (AUB). Male C57BL/6 mice (aged 6 to 8 weeks) were group-housed in a temperature-controlled room with a 12h-12h light-dark cycle and access to food and water *ad libitum*. At the time of surgery, the mice weighed between 20 and 25 g.

### Traumatic brain injury model: experimental design

Mice were initially anesthetized with Ketamine/Xylazine (50 mg/kg Ketamine and 15 mg/kg Xylazine), administered intraperitoneally. Anesthesia was then maintained using isoflurane. Following anesthesia, a midline skin incision was performed to expose the skull, and Xailin gel lubricant was applied to the eyes to protect vision during surgery. Each animal was placed on a stereotaxic frame. These mice are considered Sham mice since they did not receive any hit compared to other groups. For the repetitive mild TBI (rmTBI) group, three concussive injuries, each separated by 24 h, were conducted. For the single mild TBI (smTBI) group, a single injury was performed on the same day as the third injury for the rmTBI group. The sham group was anesthetized, the skull was exposed, and the skin incision was sutured without brain injury.

The injury was delivered using the Leica Impact One Stereotaxic Impactor (Leica Microsystems Inc., Buffalo Grove, IL, USA). This model is most used for open head injuries; however, it was modified by adding a rubber tip of 5 mm diameter to reduce the incidence of skull fracture and deliver a closed head injury. The Bregma and Lambda were pointed out to the software by Angle Two system ® (Leica Microsystems Inc.), and the target area (parietal cortex) was then localized at Bregma −1.58 mm and interaural 2.22 mm and the impact was applied at a depth of 0.3 mm, with a speed of 4 m/s, and a dwell time of 1 s. The procedure was completed with the closure of the incision using silk sutures. Each animal was then placed on a heating pad to maintain body temperature. Proteomic and behavioral tests will then be performed with these smTBI and rmTBI animals (for proteomic analysis: sham *n* = 3; 1 hit 48 h *n* = 3 and 1 week *n* = 3; 3 hits 48 h *n* = 4 and 1 week *n* = 5; for behavioral assays *n* = 10 per each group (Supplementary Fig. 1).

### Shotgun proteomics

Protein lysates were extracted using the sodium dodecyl sulfate buffer (4% SDS, Tris 0.1 M, pH 7.8), on each piece of tissue. The samples were incubated at 95 °C for 15 min and subjected to sonication for 15 min, until complete homogenization with the help of a potter. To remove DNA, the samples were centrifuged for 10 min at 16,000 × *g*. Then, the supernatant was kept, and each sample was assayed. All sample concentration was normalized to obtain a final concentration of 1 mg/ml per sample, and then processed by shotgun strategy using the FASP (Filter-Aided Sample Preparation) protocol using a filter with a cut-off threshold of 30 kDa. An equal volume of 0.1 M Dithiothreitol (DTT) reduction solution was added to each sample, and the mixture was incubated at 56 °C for 40 min. Samples were transferred to the filter FASP and the alkylation solution (Iodoacetamide IAA 0.05 M) was added followed by incubation for 20 min at room temperature. The samples were digested overnight at 37 °C, with a trypsin solution at 40 µg/ml in NH4HCO3 buffer (0.5 M).

### LC-MS/MS analysis

Samples were separated by online reversed-Phase chromatography using an EASY-nLC 1000 (Thermo Scientific) equipped with a trap column (75 μm ID × 2 cm, 3 µm, Thermo Scientific) and a C18 packed-tip column (75 µm ID × 50 cm, 2 µm, Thermo Scientific). The peptides were eluted using a gradient of ACN starting from 5% to 30% over 2 h at a flow rate of 300 nl/min. The mass spectrometer Q-Exactive (Thermo Scientific) was set to acquire data in data-dependent mode, targeting the 10 most intense ions of the MS analysis (Top 10). The MS analyses were set to a 70,000 FWHM resolution (m/z 400), with an automatic gain control target of 3e6, and a maximum injection time at 120 ms. For a full scan MS, the scan range was set between m/z 300–1600. For ddMS², the scan range was set between m/z 200–2000, 1 microscan was acquired at 17,500 FWHM, an AGC was set at 5e4 ions, and a maximum injection time of 60 ms. The isolation window was set at m/z 4.0.

### Proteomic data interrogation

All the MS/MS data were analyzed by MaxQuant (version 1.5.8.3) using the Andromeda search engine. Identification of proteins was performed by comparing all the raw spectra with a Mus musculus proteome reference from the Uniprot database (Release November 2017, 16931 entries). Trypsin specificity was used for digestion, with up to two missed cleavages. N-terminal acetylation and methionine oxidation were selected as the variable modifications. Carbarmidomethylation of cysteines was set as a fixed modification. Identification of proteins was based on at least two peptides for each protein, with one peptide being unique to each. An initial mass accuracy of 6 ppm was specified for the MS spectra, and a tolerance of 20 ppm was applied to the MS/MS fragmentation data. The false discovery rate (FDR) threshold was adjusted to 1% for protein and peptide for reliable analysis. Relative protein quantification (label-free) was conducted through the MaxLFQ algorithm incorporated into MaxQuant with the default settings.

### Bioinformatic data analyses

*Data preparation*: the proteomics dataset was pre-processed, and normalization was performed utilizing the DEP package within the R environment.^97^ The dataset initially consisted of 4851 protein identifiers, of which 321 were corresponding to contaminants, non-specific identifiers, unmappable identifiers, and duplicated protein IDs were omitted prior to normalization. Of the remaining 4530 identifiers, the ones that are not detected across all replicates within at least one condition (892in total) were excluded from the analysis (Supplementary Fig. 5a–c). After filtering, the dataset, consisting of 3638 protein IDs, went through background correction as well as normalization using variance stabilizing transformation (Supplementary Fig. 5d, e). A heatmap of proteins with missing values displayed clustering pattern based on condition (Supplementary Fig. 5f). In addition, the intensity distribution along with the corresponding cumulative fraction plots indicated that the missing values are enriched in proteins displaying low intensity levels (Supplementary Fig. 5g). These results confirm that proteins are missing not at random (MNAR), thus, the left-censoring imputation approach, MinProb, was used (Supplementary Fig. 5h). The processed dataset with log2 transformation is presented in Supplementary Table 1.

#### Fold-change analysis

DEP package was used to perform the differential expression analysis via protein-wise linear models with empirical Bayes statistics, implemented through the limma algorithm. In the pairwise analysis across the groups, the proteins displaying a Log2 Fold-Change (FC) greater than 1.5 and a BH-adjusted *p* value below 0.05 were recognized as significantly disregulated.

*Enrichment analysis*: enrichment analysis of gene ontology (GO) biological process (BP) terms was performed in R environment via topGO package (https://bioconductor.org/packages/release/bioc/html/topGO.html). The GO terms with a fold of enrichment above 2 and a weighted-Ficher *p* value below 0.05 were identified as significant. The circlize package in R environment was used to generate Circos plots displaying the top enriched terms together with their corresponding proteins.^98^

*Data visualization*: the plots of the principal component analysis (PCA) were created with the DEP package from Bioconductor in R. Heatmaps were made using Bioconductor’s ComplexHeatmap R package.^98^ Boxplots were generated using ggplot2 package in R (https://cran.r-project.org/web/packages/ggplot2/).

*Weighted gene co-expression network analysis (WGCNA)*: Signed-WGCNA was conducted to develop a protein network through topological overlap-based dissimilarity metrics using a soft-thresholding of 12. Modules with positively intercorrelated proteins were detected using the dynamic tree cutting and a deep split threshold. WGCNA package in the R environment was used to conduct the analysis.^99^ PPI networks were visualized using Cytoscape software.^100^

### Proteome discoverer 2.5

Raw LC-MS/MS data were processed using Proteome Discoverer v2.5 (Thermo Fisher Scientific) with the Sequest HT search engine. Trypsin was selected as the proteolytic enzyme, allowing up to two missed cleavages. The protein database used for identification was UniprotKB Mus musculus (17,235 sequences, Uniprot, March 2025). The search parameters included methionine oxidation and protein N-terminal acetylation as variable modifications, while carbamidomethylation of cysteines was set as a fixed modification. The minimum peptide length was set to six amino acids, with a precursor mass tolerance of 10 ppm and a fragment mass tolerance of 0.02 Da. Peptide Spectrum Matches and peptides were validated using FDR thresholds between 0.01 and 0.05. The final validation was performed using Percolator, applying strict FDR = 0.01 and relaxed FDR = 0.05. Proteins were considered identified if at least two peptides matched per sequence. Protein quantification analysis was performed using the Quan Rollup workflow. Protein abundances were calculated using the summed abundances approach. For quantification, the Top N parameter was set to 3, and pairwise ratio-based calculations were applied to determine protein ratios. The maximum allowed fold change was set to 100. No imputation was applied to missing values.

### Data-independent acquisition LC–MS/MS analysis

Desalting of each sample was carried out using an Evotip Pure (Evosep, Denmark) according to the manufacturer’s protocol. Analysis of the peptides was conducted using an Evosep One liquid chromatography (LC) system coupled to a timsTOF HT mass spectrometer (Bruker Daltonics, Germany). The Evosep One system was configured to run under the 60 Samples Per Day (60 SPD) method, equipped with a C18 performance column (EV1109; 8 cm × 150 μm, 1.5 μm particle size) and maintained at 40 °C. The analytical column was coupled to a fused silica emitter (10 μm inner diameter, Bruker Daltonics) integrated into a CaptiveSpray source (Bruker). Data acquisition was carried out in DIA-PASEF mode, and spectra recorded across an m/z range of 100–1700 with an ion mobility range of 1/K0 = 1.51 V · cm^−2^ to 1/K0 = 0.6 V · cm^−2^.

### DIA-NN raw data analysis

Raw data were analyzed using DIA-NN* software (version 1.9.2). A library-free workflow was employed to check against the UniProt-reviewed Homo sapiens database (September 2024, 20,420 entries). Search parameters allowed up to two missed cleavages for trypsin digestion, with methionine oxidation specified as a variable modification. Peptides were filtered using a length range of 7–30 amino acids, and a precursor charge range of 2–4, whereas the precursor m/z range was between 300–1300, and a fragment ion m/z range of 100–1700. The FDRs were set to 1% at both the protein and peptide levels. “Match between runs” was enabled, and the quantification strategy was set to “robust LC (high accuracy).” The identified proteins were subjected to further analysis using Perseus software (version 1.6.10.43). For comparisons between all groups, statistical analysis was conducted with the Multiple sample test and a *p* value of 0,001. Data were normalized using Z-scores, and only statistically significant proteins were included in hierarchical clustering analyses.

### Machine learning data analysis

Unsupervised, supervised learning, and predictions explanation were implemented using Python 3.11.9. The matrix data was imported into Python using the Pandas library (version 2.0.3) and structured as a DataFrame.

For unsupervised learning, the t-distributed Stochastic Neighbor Embedding (t-SNE) algorithm from the Scikit-learn library (version 1.2.2) was employed. t-SNE is a nonlinear dimensionality reduction technique particularly well-suited for high-dimensional biological data, as it captures complex relationships and preserves local structure while emphasizing the separation of distinct clusters. The algorithm reduces the data to two dimensions (tsne1 and tsne2), enabling a clearer visualization of sample grouping. The perplexity parameter was set to 10, balancing sensitivity to local and global structures in the data. A lower perplexity value emphasizes local relationships, making it suitable for datasets with small to medium sample sizes. To ensure reproducibility, the random state was set to 1. All visualizations were generated using the Matplotlib library (version 3.7.2), allowing for a clear representation of the t-SNE projections and their clustering patterns.

For the supervised learning, the Lazy Predict library (https://lazypredict.readthedocs.io/en/latest/) was used to build multiple classification models from the scikit-learn library by training and testing a range of 24 classifiers. The random state was always kept at 1. Subsequently, the optimal model was reconstructed individually using the scikit-learn library, which facilitated the adjustment of its parameters to optimize and assess its accuracy. A 5-fold cross-validation was carried out to evaluate the performance of the model using KFold and cross_val_score functions, with the classification report generated through the classification_report function. The Confusion_Matrix_Display function from the matplotlib library was further employed to plot the confusion matrix.^101^

The LIME algorithm was used to explain the decision-making and predictions of the model. The LIME algorithm calculates feature contributions (proteins) that can be either positive or negative. The ELI5 library (https://eli5.readthedocs.io/en/latest/overview.html) was employed to create a LIME table that includes the weight of feature contributions through the explain_prediction function^102^ To further enhance interpretability, we visualized the top contributing proteins using a bubble plot created with the Plotly library (version 5.17.0). This visualization not only highlights key protein biomarkers but also provides an intuitive representation of their relative importance across different conditions. By integrating these explainability methods, we mitigate the “black-box” nature of the model, ensuring that predictions are interpretable and biologically meaningful.

### Behavioral assessment

Behavioral assessments were conducted to evaluate motor performance, cognitive function, and anxiety-like behavior. Mice were divided into three groups: sham (*n* = 10), single mTBI (*n* = 10), and repetitive mTBI (*n* = 10) groups. Male C57BL/6 mice (6–8 weeks old, ~20–25 g weight) were acclimated to experimenters and experimental rooms for two weeks before surgery and behavioral assessment. After the injury (smTBI and rmTBI), neurological and cognitive tests were assessed: *Pole climbing test, grip strength test, Morris water maze test, and elevated plus maze test*. The mice were sacrificed 2 days, 7 days, or 30 days post-injury representing acute, sub-acute, and chronic stages, respectively.

### Pole climbing test

The pole-climbing test was performed to evaluate the ability of the mice to descend a vertical metal rod (60 cm height, 1 cm diameter) set perpendicularly in an empty box. Animals were habituated on the testing days to descend on the pole for three trials before the recorded testing. The mice were placed on top of the pole with their heads directed upwards and were then allowed to descend freely. The time needed for each mouse to reach the bottom of the pole (t-total) was recorded. For each animal, three testing trials were done on each testing day before sacrifice at 2, 7, and 30 days following the injury.

### Grip strength test

The 4700 grip strength meter (UGO BASILE, Gemonio, Italy) was used to assess muscle strength after TBI. Each animal is held by its tail and is allowed to catch a trapeze-shaped metal with both of its paws. A total of three trials were executed for each animal on testing days. Muscle strength in gram force (gf) was registered. For analysis, the average of muscle strength, in gf, in the three trials, was normalized to the weight of the mouse.

### Morris water maze test

The Morris Water Maze (MWM) test is performed to assess spatial learning and memory function in rodents.^15^ MWM test was employed during the last 5 days before sacrifice for the subacute (day 7) and chronic (day 30) time points. The apparatus mainly consists of a circular pool (0.55-m depth and 1-m diameter), half-filled with opaque water (maintained at 25–26 °C) by adding non-toxic white paint to ensure accurate camera tracking and prevent water reflection. This experiment was not executed in the day 2 group because it requires at least 6 days to complete.

MWM comprises learning (platform is visible), spatial learning (invisible platform with spatial cues), and memory trials (platform is removed with spatial cues). Three cues of different shapes were placed on three walls surrounding the pool, where their reflection guides the mice spatially to a transparent escape platform of 30 cm height and 10 cm diameter placed in the mid-North East (NE) quadrant, with water covering the top. To monitor the movement of the mice and capture other parameters, the ANY-maze 5.2 software (Stoelting Co., Wood Dale, Illinois, USA) was employed. For five consecutive days, mice were subjected to three trials on each testing day, except for the 5th day. The platform was fixed in the NE quadrant, the target quadrant. On the first day, the platform was made visible by a flag placed on the platform, which ensures that any latency differences during the following testing days are due to memory deficits but not impaired eyesight. This was followed by learning trials, which were done for four consecutive days including the 1st day, where the flag was removed, the platform was submerged and hidden in the water, and the animals were expected to locate the platform. The starting position of animals varied among trials. The latency to reach the platform was recorded. On day five, the platform was removed, and the mice were allowed to swim for 60 s for one trial only, known as the probe trial. The time spent in each quadrant and the latency to reach the NE target quadrant were recorded. Throughout the test, after each trial, the mice were dried with a towel and allowed to rest in a heated cage.

### The elevated plus maze test

The elevated plus maze test (EPM) evaluates the fear and anxiety-like behaviors 7 and 30 days after injury. The maze consists of a platform elevated 40 cm from the floor with four arms intersecting at a 90° angle, and each arm is 35 cm long and 5 cm wide. Two arms are enclosed to make the “dark arms” while the other two arms are kept open to form the “open arms”. Mice with anxiety-like behavior usually have a preference for dark enclosed arms over bright open arms. The test is executed only once for each mouse, and it is initiated by placing the mouse at the intersection point of these arms with its head facing one of the open arms. Each mouse was left to explore the maze for 5 min. The time spent and the number of entries into the open and dark arms were tracked using the video camera positioned over the maze and analyzed using ANY-maze 5.2 software.

### Behavioral statistical analysis

All analyses were performed using Python (v3.11.3), specifically the pandas (v1.5.3), seaborn (v0.13.2), matplotlib (v3.7.1) and statsmodels (v0.13.5) libraries. A total of 10 animals were used for each behavioral test. Behavior data were analyzed with linear mixed-effects models (LMM) in Python, including group and time as fixed effects and animal as a random effect. Mixed linear models were fitted using the MixedLM function from statsmodels. Graphs were generated with seaborn.pointplot and matplotlib.pyplot, with custom styling for clarity. Behavioral data were structured in long-format tables with the following columns: Mouse_ID, Test_Type, Day, Group, and Measurement, along with test-specific factors such as Arm_Type for the EPM. Each mouse was uniquely identified and tested across different time points to allow repeated measures analysis. In the MWM the analyzed parameters included: latency to platform (escape latency), latency in the NE quadrant, percentage of time spent in the NE quadrant, over multiple days (Day 1 to Day 4) at 7- and 30-days post-injury. Therefore, a linear mixed-effects model was applied to each outcome variable across training days with Group (sham, smTBI, and rmTBI) as a fixed effect and Mouse_ID as a random effect to account for repeated measures within animals. For the EPM, data were collected at 7- and 30-days post-injury. Separate mixed-effects models were fitted for each arm type and each time point, with Group as a fixed effect and Mouse_ID as a random intercept. Pole climbing performance was measured at 2, 7, and 30 days following injury and the time required for each mouse to descend the pole was recorded. A mixed-effects linear regression was used to model the effect of Group on performance at each time point, including Mouse_ID as a random effect. Grip strength was evaluated using a digital grip strength meter at 7- and 30-days post-injury. A separate mixed-effects model was fitted for each day, with Group as a fixed effect and Mouse_ID as a random intercept, to assess muscle strength deficits across conditions (Supplementary Figs. 9, 10).

Mixed linear models were selected to appropriately account for within-subject repeated measures over time. Model results included estimated coefficients, standard errors, z-scores, *p* values, and 95% confidence intervals. Convergence diagnostics and variance components of the random effects were verified for all model fits. A *p* < 0.05 was considered statistically significant (*p < 0.05, **p < 0.01, ***p < 0.001, ****p < 0.0001, ns: *p* > 0.05).

## Supplementary information

Supplemnatary Materials

Supp. Table 1

Supp. Table 2

Supp. Table 3

Supp. Table 4

Supp. Table 5

Supp. Table 6

Supp. Table 7

Supp. Table 8

Supp. Table 9

Supp. Table 10

Supp. Table 11

## Data Availability

The raw data and result files used for analysis were deposited at the ProteomeXchange Consortium.^103^ (http://proteomecentral.proteomexchange.org) via the PRIDE partner repository with the dataset identifier PXD011418.
