# Supplementary material for: Uncovering injury-specific proteomic signatures and neurodegenerative risks in single and repetitive traumatic brain injury
Source: Signal Transduct Target Ther. 2025 Jun 23;10:195. doi: 10.1038/s41392-025-02286-9 (PMC12183312; doi:10.1038/s41392-025-02286-9)
Supplement: Supplementary file 1 — Supplemnatary Materials [file 41392_2025_2286_MOESM1_ESM.docx]

Supplementary Materials for

Uncovering injury-specific proteomic signatures and neurodegenerative risks in single and repetitive traumatic brain injury

Sarah Mantash1*, Soulaimane Aboulouard2*, Hassan Dakik3*, Yanis Zirem2 Lydia Ziane-Chaouche2, Ali Nehme4, Khalil Mallah5, Marya El-Kurdi4, Naify Ramadan4, Isabelle Fournier2, Kazem zibara4, Firas Kobeissy4,6¥, Michel Salzet2¥

Correspondence to: Michel Salzet ([Michel.salzet@univ-lille.fr](mailto:Michel.salzet@univ-lille.fr)), Firas Kobeissy ([firasko@gmail.com](mailto:firasko@gmail.com))

**This PDF file includes:**

Figures. S1 to S10

Tables S1 to S11 (with separated files)


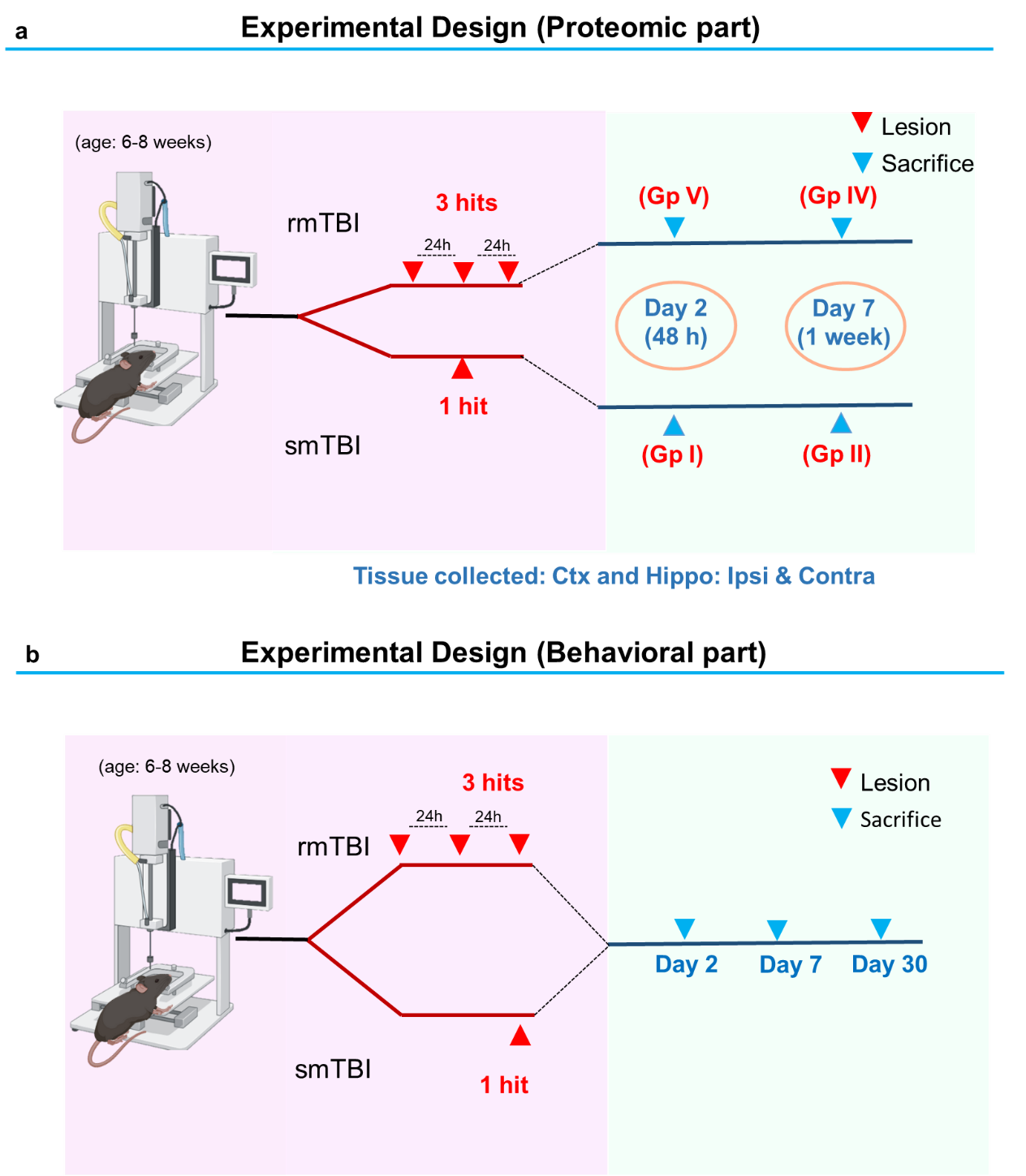


Figure S1. Experimental design for a) proteomic and b) behavior assay used. Male C57BL/6 mice (6-8 weeks old, ~20-25 g weight) were subjected to single or repetitive (3 hits) mTBI. The mice were sacrificed 2 days, 7 days, or 30 days following the injury, representing acute, sub-acute, and chronic Stages, respectively. For proteomic analysis: sham n=3; 1 hit 48h n= 3 and 1week n=3; 3 hits 48h n= 4 and 1 week n= 5; for behavioral assays n= 10 per each group.


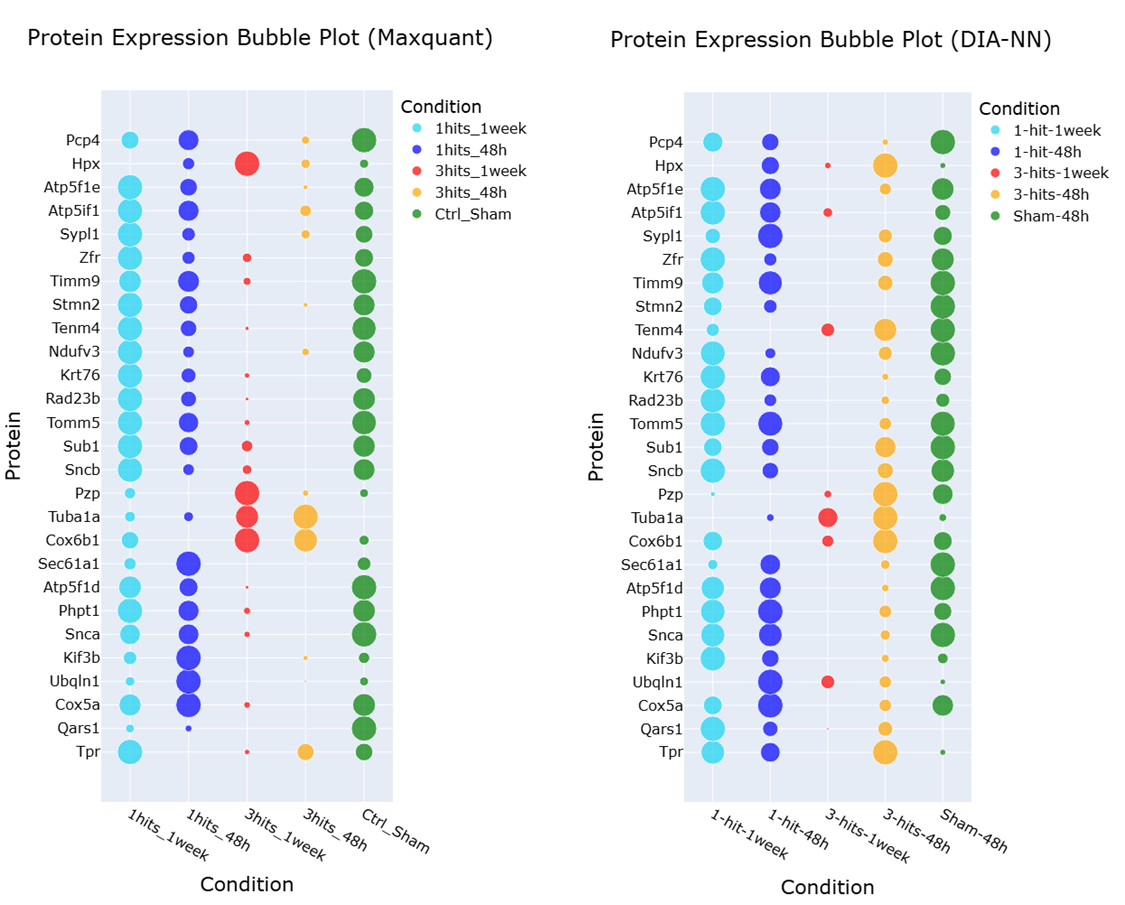


**Figure S2.** Bubble Plot of protein expression per hit and time in DDA with ML and in DIA-NN.

**
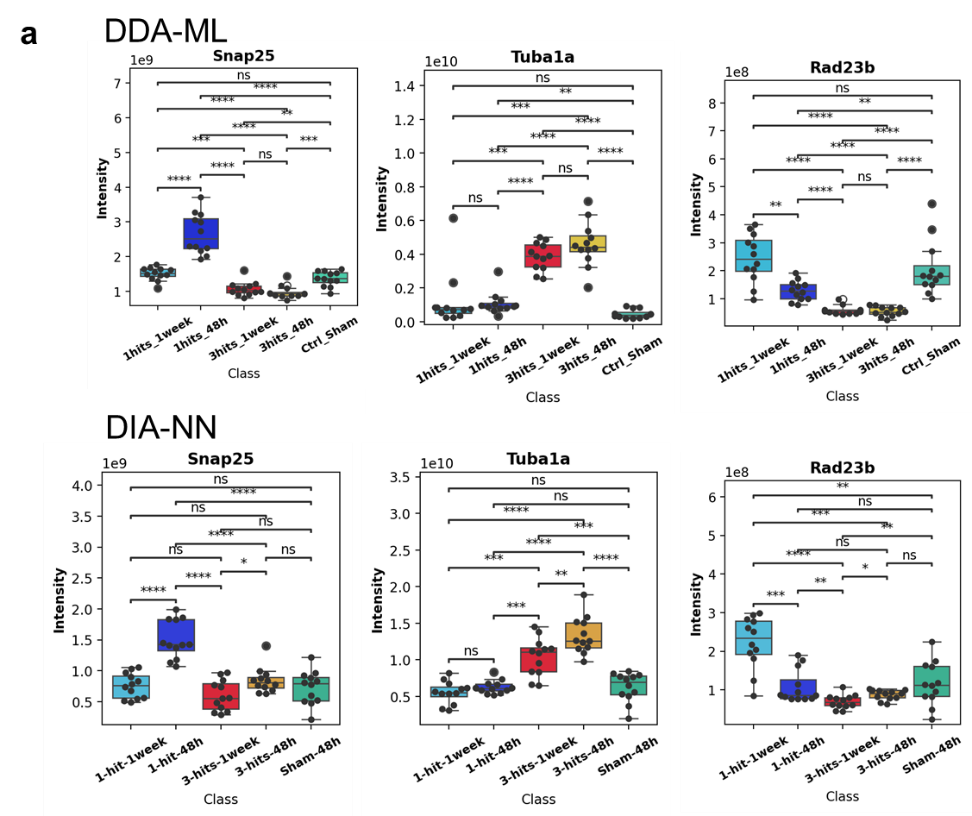
**

**
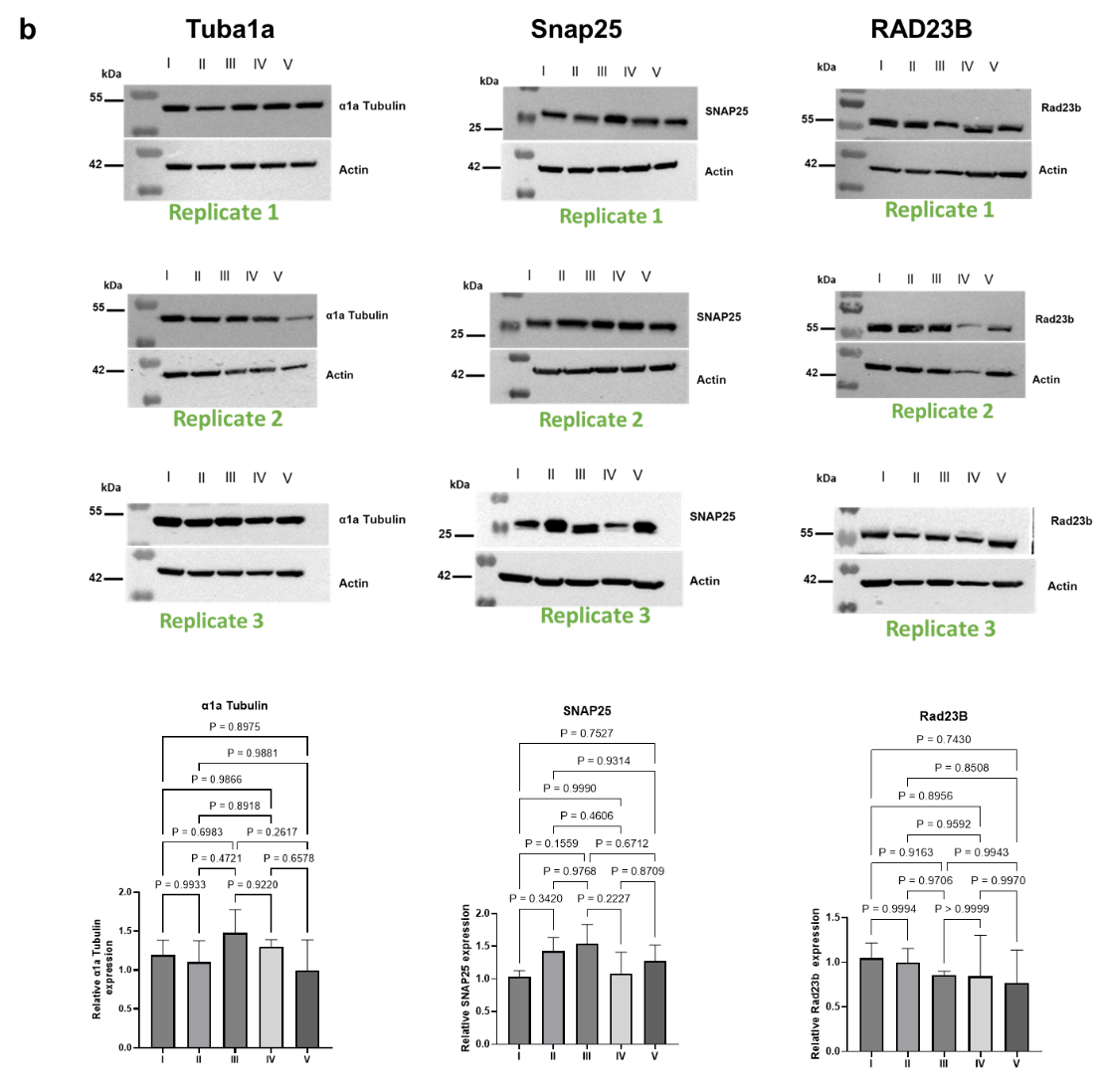
**

**Figure S3. a)** Box plot of examples of quantified proteins using DDA with ML and DIA-NN are presented with **b)** western blot analyses of Tuba1a, Snap25 and Rad23c.

**
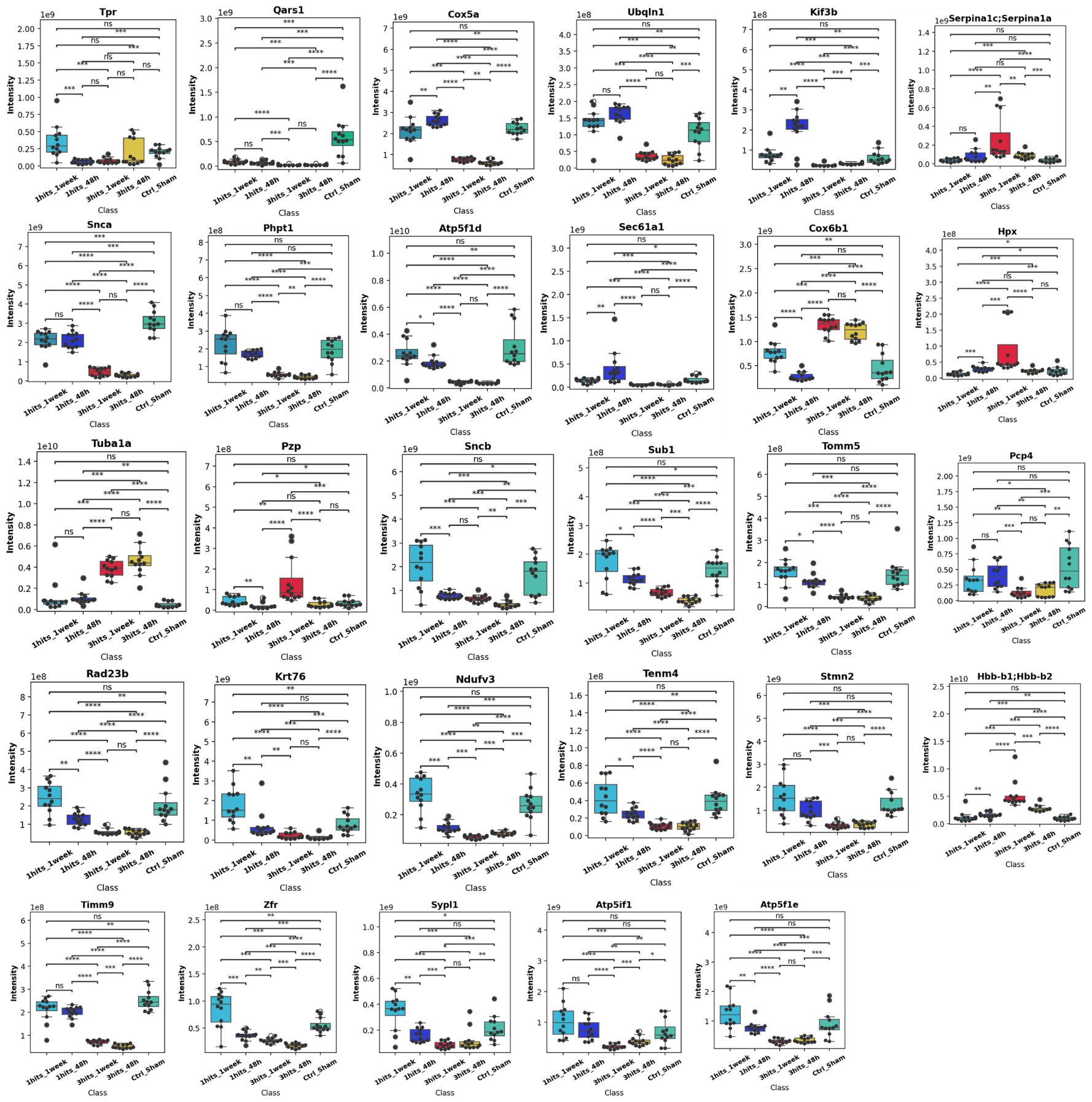
**

**Figure S4.** Box plot of quantified proteins using Data Dependent analysis (DDA) with Machine Learning (ML).

**
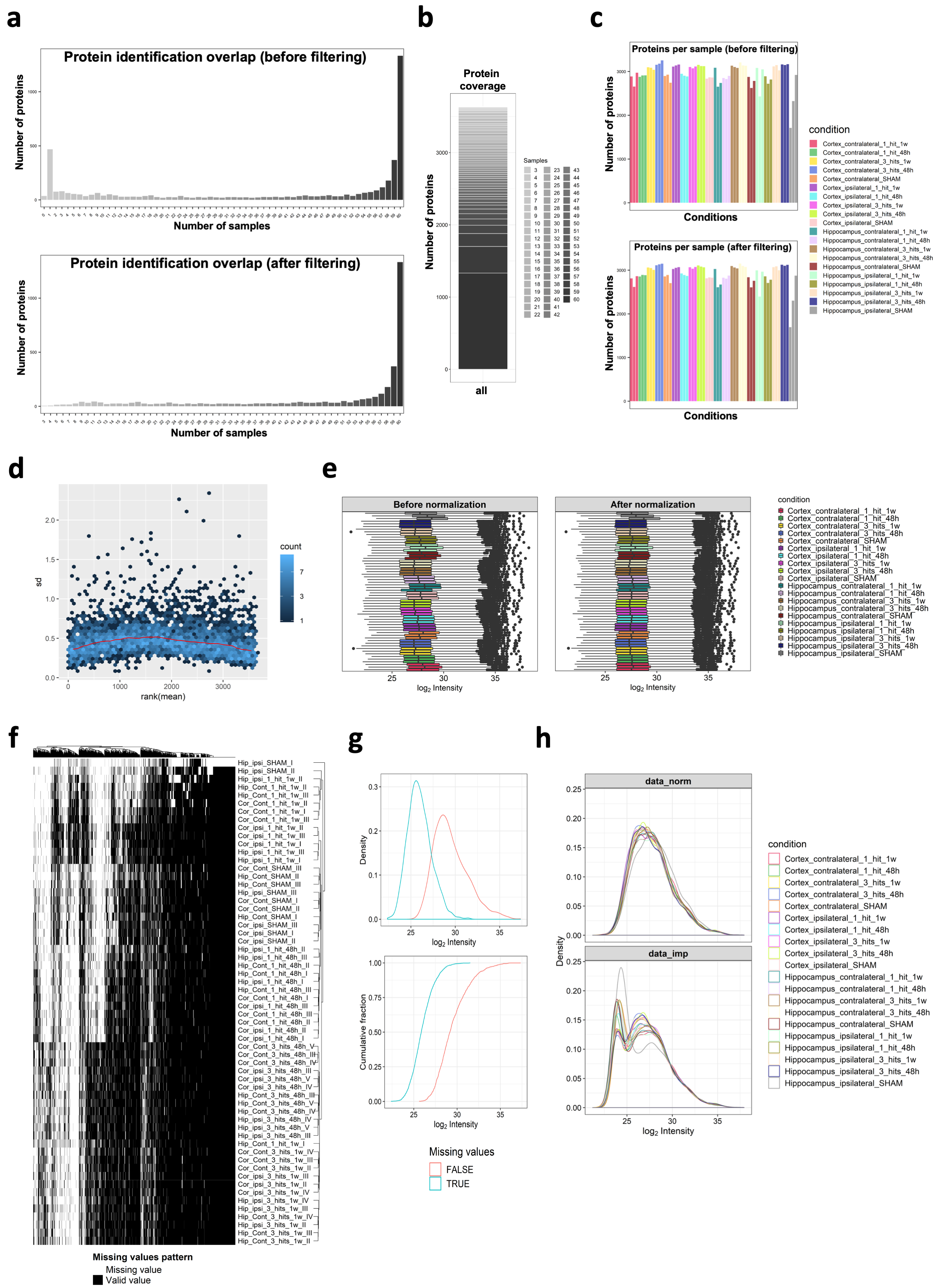
**

**Figure. S5. Data preparation and quality control.** **a**) Protein identification overlaps between samples before and after removing proteins that are absent from all replicates of at least one condition. **b**) Total number of detected proteins per sample before and after filtering. **c**) protein coverage across samples. Almost half the proteins remaining after filtering are detected in all samples, while the other half is absent from at least one sample. **d**) The dataset was background corrected and normalized by variance stabilizing transformation (VSN), which assumes that most genes are not differentially expressed, and the running median of the standard deviation (red line) must follow a horizontal line. **e**) boxplot of data distribution before and after normalization for each sample. **f**) A heatmap of the presence status for proteins with at least one missing value shows a clustering pattern by condition. **g**) Intensity distribution and cumulative fraction plots for the two protein groups: with or without missing values. **h**) Intensity distribution of proteins from all samples before and after imputation of missing values.


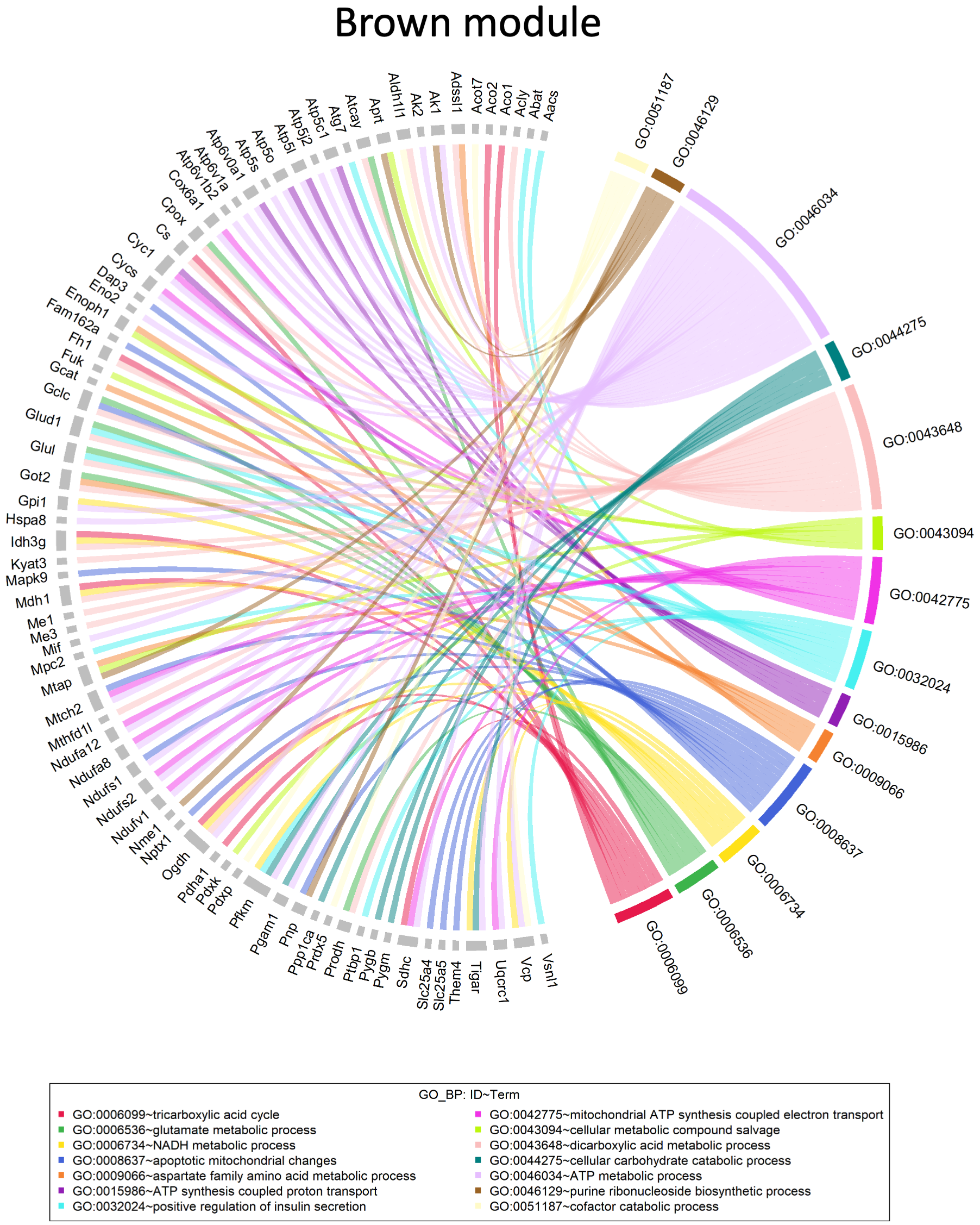


**Figure. S6. Circos plot** displaying GO biological process (BP) terms that are significantly enriched in brown-module proteins.


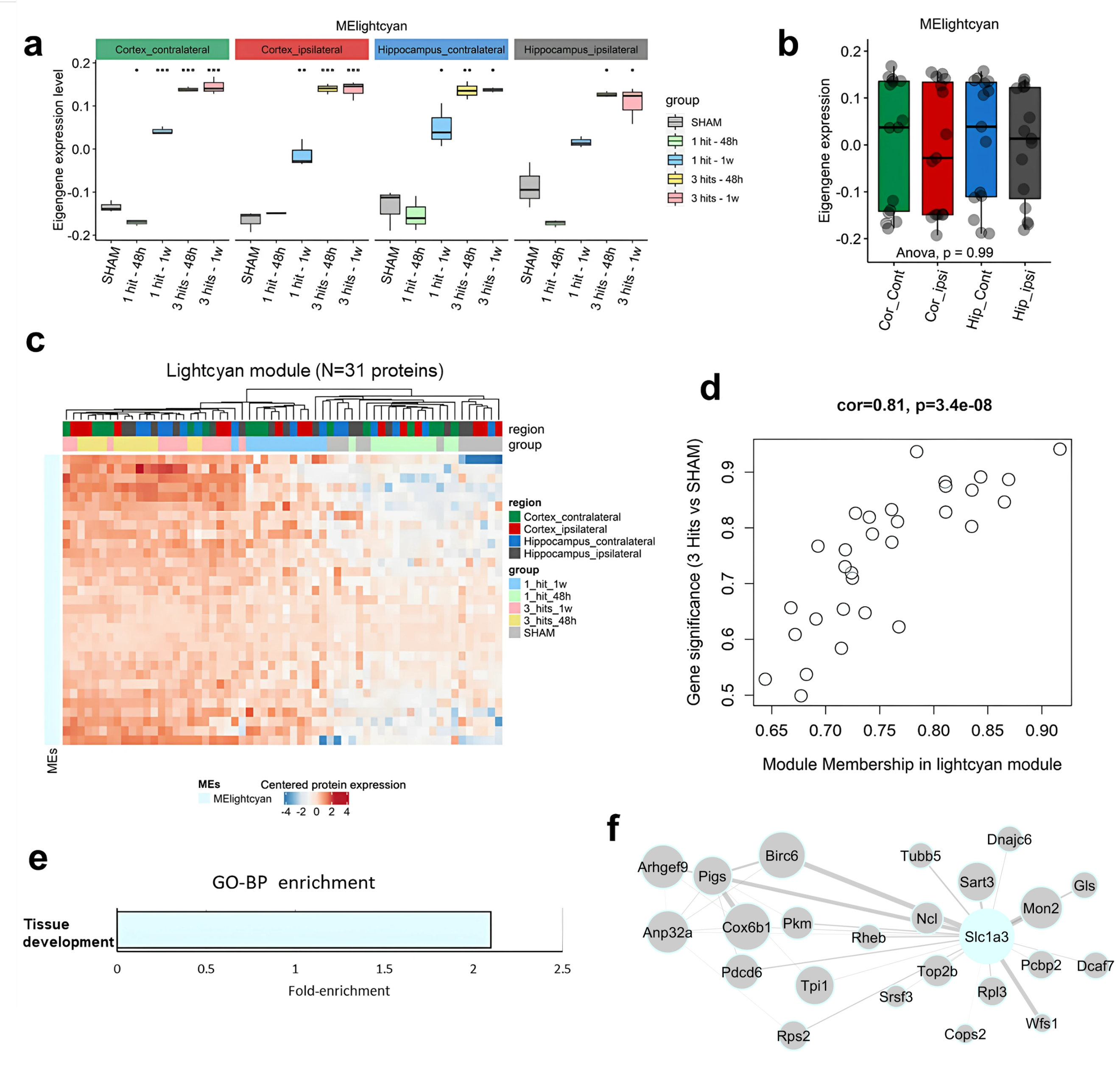


**Figure. S7. Analysis of the light cyan module**. **a**) Boxplot showing the variation of the light cyan module’s ME (ME light cyan) across SHAM and various Hit-Time conditions for each tissue. Student’s t-test was used to compare various conditions to SHAM. (*: p<0.05; **: p<0.01). **b**) Boxplot illustrating the variation of ME light cyan value between tissues. ANOVA was used for multiple comparisons. **c**) Heatmap showing the expression profile of the 31 proteins found in the light cyan module. **d**) Scatter plot showing the correlation between Gene significance (GS) and Module membership. Gene significance (GS) was defined as the correlation between protein expression and 3-hits vs SHAM, regardless of tissue of origin. Module Membership (MM) was defined as the correlation between protein expression and module eigengene. The higher the GS score the more a protein is increased by 3-hits compared to SHAM. The higher the MM score, the more a protein is interconnected within the module. A positive correlation between GS and MM (r=0.81, p=3.4x10-8) demonstrates that light cyan module constitutes an interconnected network of proteins that is increased by 3-hits. **e**) GO biological process enrichment results for proteins found in the light cyan module. Enriched proteins belong to tissue development GO term and include: Birc6, Cops2, Pdcd6, Pkm, and Rheb proteins. **f**) protein-protein interaction network showing the top interconnected proteins in the light cyan module (weighted correlation > 0.812). Node size and edge width are, respectively, proportional to module membership (MM) and weighted correlation. Hub proteins with MM score above 0.9 are colored in light cyan.

**
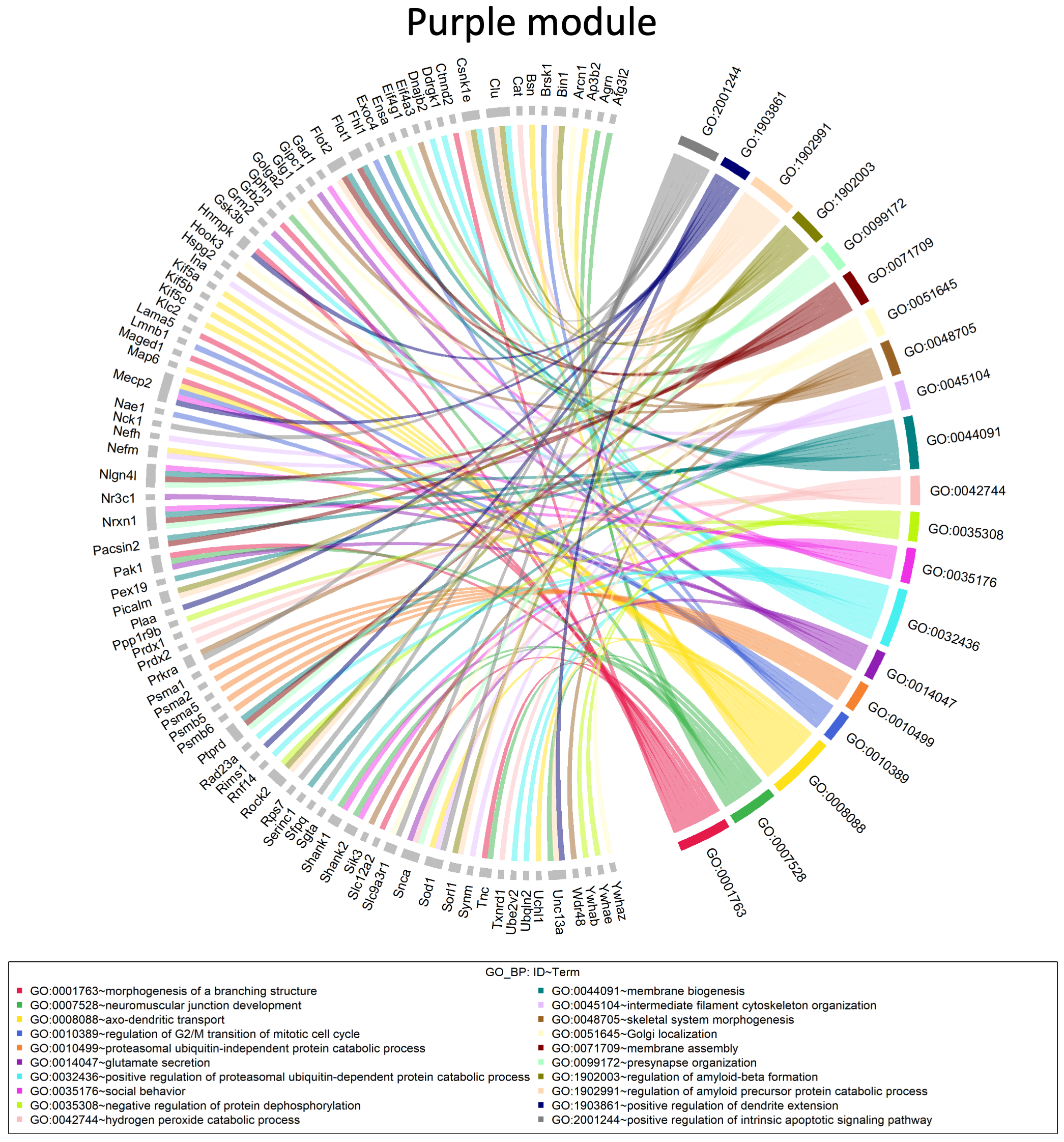
Figure. S8. Circos plot** displaying GO biological process (BP) terms that are significantly enriched in purple-module proteins.

**
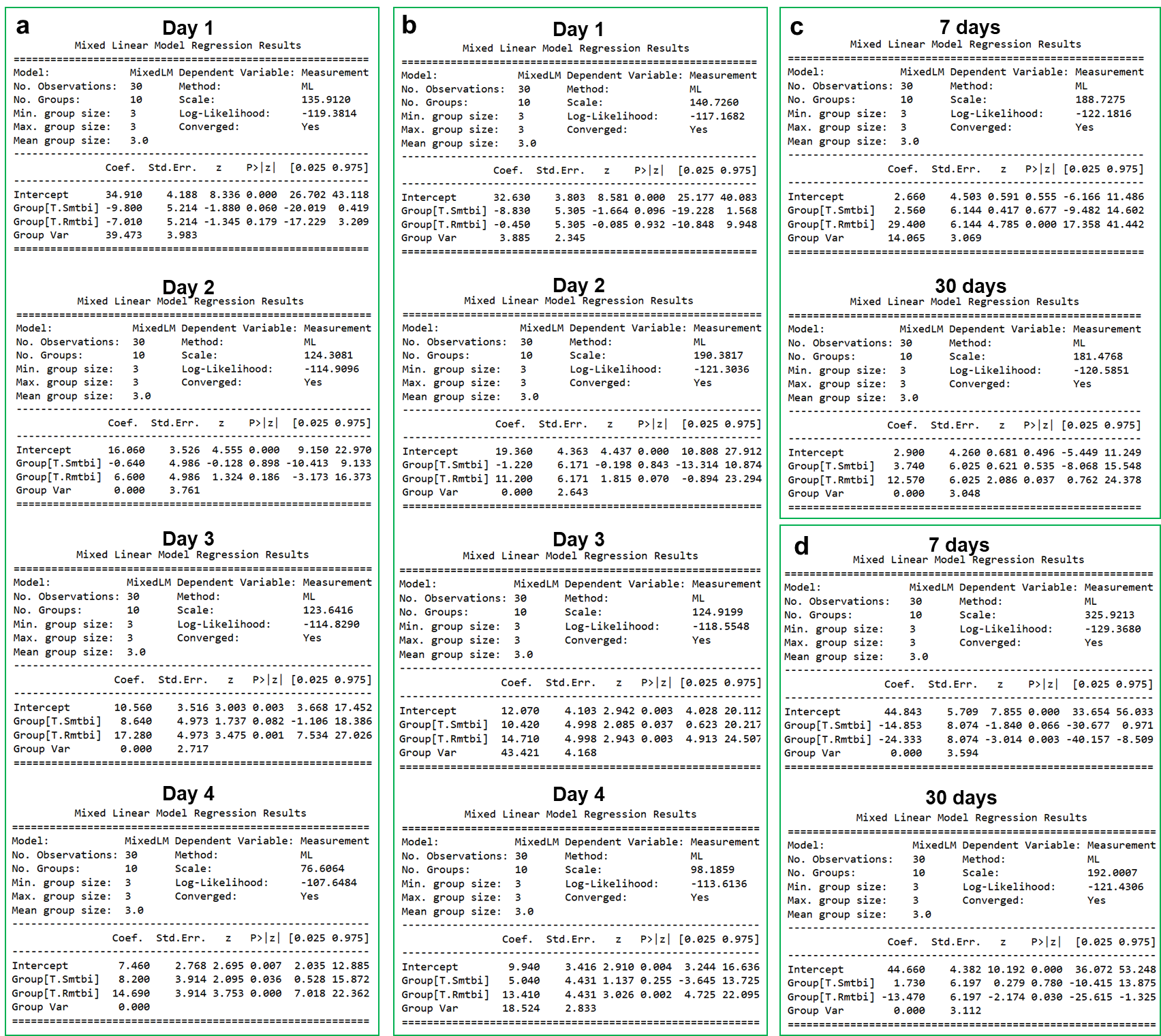
**

**Figure. S9.** Summary of the linear mixed-effects models (LMM) performed for Morris water maze tests (MWM). Escape latency over multiple days, at (**a**) 7 days and (**b**) 30 days post injury. (**c**) Latency in the NE quadrant and (**d**) Percentage of time spent in the NE quadrant, at 7 and 30 days post injury.

**
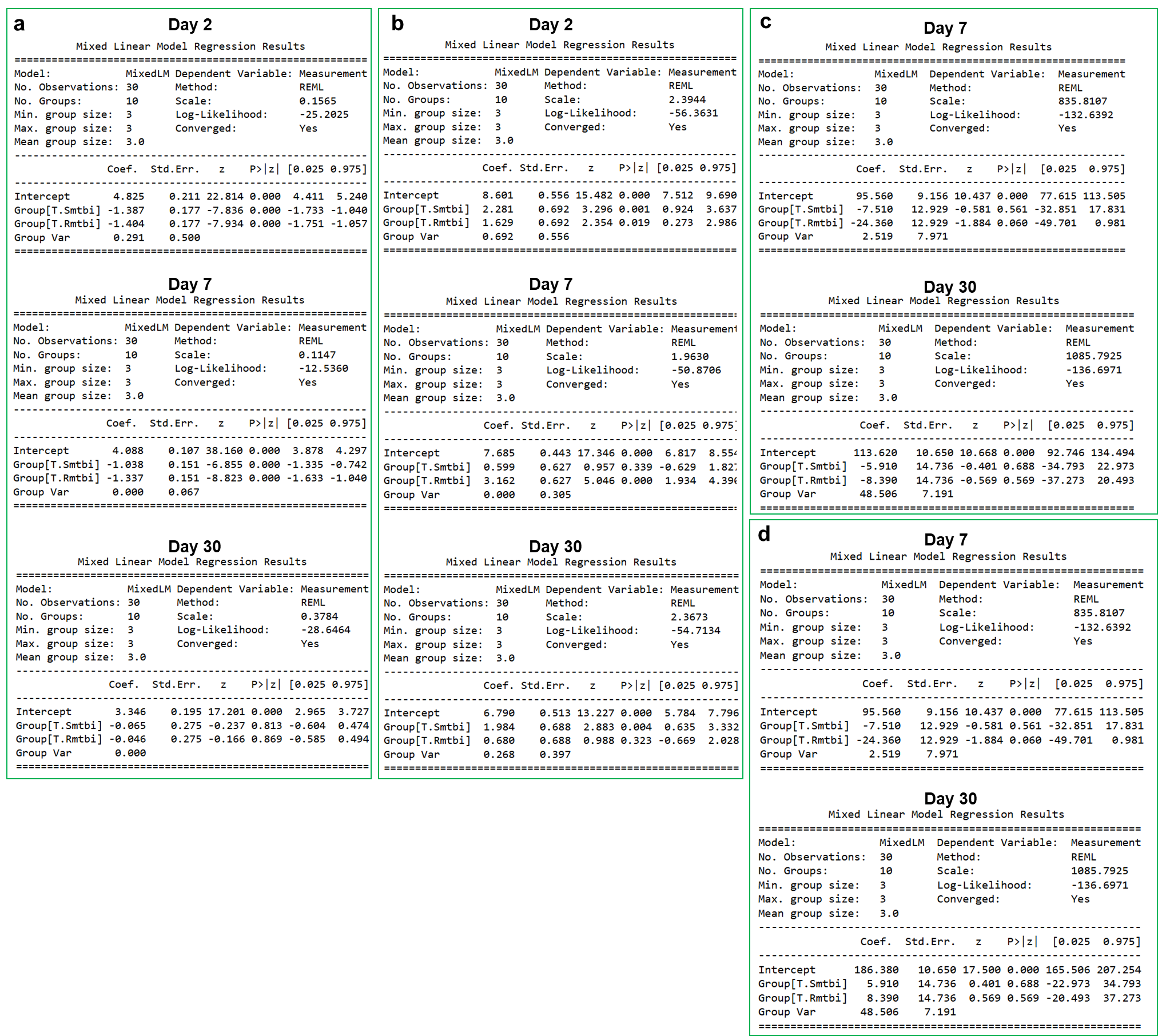
**

**Figure S10.** Summary of the linear mixed-effects models (LMM) applied to the two behavioral test paradigms. (**a**) Grip strength test and (**b**) Pole climbing test as part of the neurological assessments; time spent in the (**c**) open arms and (**d**) closed arms of the Elevated Plus Maze (EPM) test.

Separate files:

Table S1. Proteomic dataset of all conditions for smTBI and rmTBI.

Table S2. Differentially expressed proteins per condition.

Table S3. Clusters obtained in all conditions.

Table S4. Gene Ontology biological process (GO-BP) enrichment for up- and downregulated proteins for all comparisons across tissues.

Table S5. Intersection analysis of Differentially Expressed protein lists across time.

Table S6. Gene Ontology biological process (GO-BP) enrichment of timepoint-specific and time-independent DEPs.

Table S7. Intersection analysis of Differentially Expressed protein (DEP) lists across hit conditions.

Table S8. Gene Ontology biological process (GO-BP) enrichment of hit-frequency-specific and frequency-independent of Differentially Expressed protein (DEP).

Table S9. Gene Ontology biological process (GO-BP) enrichment for proteins consistently deregulated by 3 Hits across 3 tissues.

Table S10. Module membership (MM) and Gene Significance (GS) for brown, light cyan, and purple modules.

Table S11. Behavioral raw data of the pole climbing test, grip strength test, Morris water maze test (MWM), and elevated plus maze (EPM) test.
